# Immunoregulation by IL-7R-targeting antibody-drug conjugates: overcoming steroid-resistance in cancer and autoimmune disease

**DOI:** 10.1038/s41598-017-11255-4

**Published:** 2017-09-06

**Authors:** Masahiro Yasunaga, Shino Manabe, Yasuhiro Matsumura

**Affiliations:** 1 Division of Developmental Therapeutics, Exploratory Oncology Research & Clinical trial Center, National Cancer Center, 6-5-1 Kashiwanoha, Kashiwa, Chiba 277-8577 Japan; 2 Synthetic Cellular Chemistry Laboratory, RIKEN, 2-1 Hirosawa, Wako, Saitama 351-0198 Japan

## Abstract

Steroid-resistance is a common complication in the treatment of malignancies and autoimmune diseases. IL-7/IL-7R signaling, which regulates lymphocyte growth and survival, has been implicated in the development of malignancies and autoimmune diseases. However, the biological significance of IL-7/IL-7R signaling in steroid treatment is poorly understood. Here, we identified a novel relationship between IL-7R signaling and steroid-resistance, and showed that an anti-IL-7R antibody conjugated with SN-38 (A7R-ADC-SN-38) has strong anti-tumor effects against both parental and steroid-resistant malignant cells. Furthermore, inflammation in the mouse autoimmune arthritis model was suppressed to greater extent by A7R-ADC conjugated to MMAE than by A7R-ADC-SN-38. Given that an increased proportion of IL-7R-positive cells is a common mechanism underlying the pathogenesis of autoimmunity, we found that specific depletion of this cell population abrogated the progression of disease. This suggests that the cytotoxicity and immunosuppressive capacity of A7R-ADC could be modulated to treat specific malignancies or autoimmune diseases through the introduction of different payloads, and represents a novel alternative to steroid therapy.

## Introduction

In the cancer moonshot strategy, more insight into the mechanisms regulating immune homeostasis in health and disease has been required to develop new immunotherapies^1^. However, there are several concerns regarding the control of immune reactions to treat malignancies. The most popular example may be the recent use of anti-CTLA-4 and anti-PD-1/PD-L1 antibodies as immune checkpoint blockades. Although these treatments can induce significant anti-tumor effects by enhancing immune reactions, unique adverse effects involving the development of autoimmune diseases such as arthritis, dermatitis, colitis, pneumonitis, hepatitis and hypophysitis have been simultaneously observed^2^. Thus, the cross-disciplinary study of malignancy and autoimmune disease has become very important.

Steroids are commonly used in the treatment of lymphoid malignancies (leukemia and lymphoma) and autoimmune diseases. Although steroids are major physiological regulators of the immune system and provide substantial clinical benefits, they affect homeostasis in the whole body. Several adverse effects such as neuropsychological impairment, metabolic disturbance or secondary osteoporosis can lead to the discontinuation of the treatment^3^. Steroid resistance is another important component in the clinical management of patients with lymphoid malignancies and autoimmune diseases^4–6^. Novel immunoregulatory treatments that serve as an alternative to steroids or are able to overcome steroid-resistance have been strongly desired.

Intriguingly, excessive IL-7/IL-7R signaling, which otherwise regulates lymphopoiesis and promotes B- and T-cell proliferation and survival^7^, has recently been shown to contribute to the progression of lymphoid malignancies^8, 9^. Physiologically, IL-7/IL-7R signaling plays a key role in the development and remodeling of lymph nodes (LNs)^10, 11^. While blocking this signaling causes severe lymphopenia^12–14^, a gain-of-function mutation in IL-7R has been shown to act as an oncogene in approximately 10% of T-cell acute lymphoblastic leukemias (ALLs) and 1% of B-cell ALLs^8, 15^. Several authors have also reported that IL-7R expression in lung, breast or prostate cancer cells is associated with tumor aggressiveness, lymphovascular invasion and lymphangiogenesis^16–18^. Therefore, IL-7R targeting might provide a new paradigm for the development of novel therapies to treat both lymphoid malignancies and metastatic solid tumors.

IL-7/IL-7R signaling also physiologically regulates the selection of antigen-reactive T cells^19–21^. Therefore, aberrant IL-7/IL-7R signaling has been implicated in the pathogenesis of various autoimmune or inflammatory diseases such as multiple sclerosis, type 1 diabetes mellitus, rheumatoid arthritis and ulcerative colitis^8, 22–25^. Moreover, anti-IL-7R-neutralizing monoclonal antibodies (mAbs) have been shown to be effective in preclinical studies of autoimmune diseases^23, 24, 26^. Thus, IL-7R targeting, perhaps through mAbs, might be a means of treating both lymphoid malignancies and autoimmune diseases. However, there is no clear evidence as yet of an anti-tumor effect of such mAbs against lymphoid malignancies or solid tumors, and ligand-independent constitutive IL-7R signaling or autoactivation of downstream pathways may abrogate any antibody-dependent neutralizing effect. In addition, the efficacy of an anti-IL-7R neutralizing mAb was insufficient to control the inflammation of autoimmune arthritis in mice^26^. To overcome these drawbacks, a new approach is required.

Antibody-drug conjugates (ADCs) are next-generation antibody therapeutics that have shown strong anti-tumor effects against metastatic or remnant refractory cancers^27^. These compounds deliver highly toxic anticancer agents (ACAs) to and selectively eliminate tumor cells^27^, as demonstrated by an anti-HER2 ADC that was effective against target cells, even when patients had therapeutic resistance against anti-HER2 antibodies^28^. Thus, we hypothesized that ADCs targeting IL-7R would be effective against lymphoid malignancies, even if IL-7R signaling was disrupted by ligand independence or autoactivation of downstream signaling pathways.

We observed IL-7R expression in both malignant lymphoid cells and metastatic solid tumor cells and found that abrogation of this expression reduced tumor aggressiveness. IL-7R-dependent steroid-resistance was also observed, but only in malignant lymphoid cells. This resistance did not appear to be dependent on the NR3C1 steroid receptor, BCL2, or the JAK/STAT or PI3K/AKT pathway, nor did our data support a role for NF-κB activation in promoting IL-7R-dependent steroid-resistance. Furthermore, IL-7R-positive bone marrow B cells and splenic T cells were resistant to steroid therapy *in vivo*. In the context of autoimmune disease, IL-7R^high^-positive lymphocytes were increased at sites of uncontrolled inflammation in a collagen antibody-induced arthritis (CAIA) mouse model. Having confirmed a relationship between IL-7R signaling and steroid-resistance, we developed an anti-IL-7R antibody ADC (A7R-ADC). This ADC showed significant anti-tumor and anti-inflammatory effects against both lymphoid malignancy and autoimmune arthritis and was able to target both steroid-sensitive and steroid-resistant cells. Thus, A7R-ADC may be a novel, promising alternative to current treatments for these diseases.

## Results

### IL-7R signaling promotes tumor aggressiveness in both malignant lymphoid and metastatic solid tumor cells

**Figure 1:**
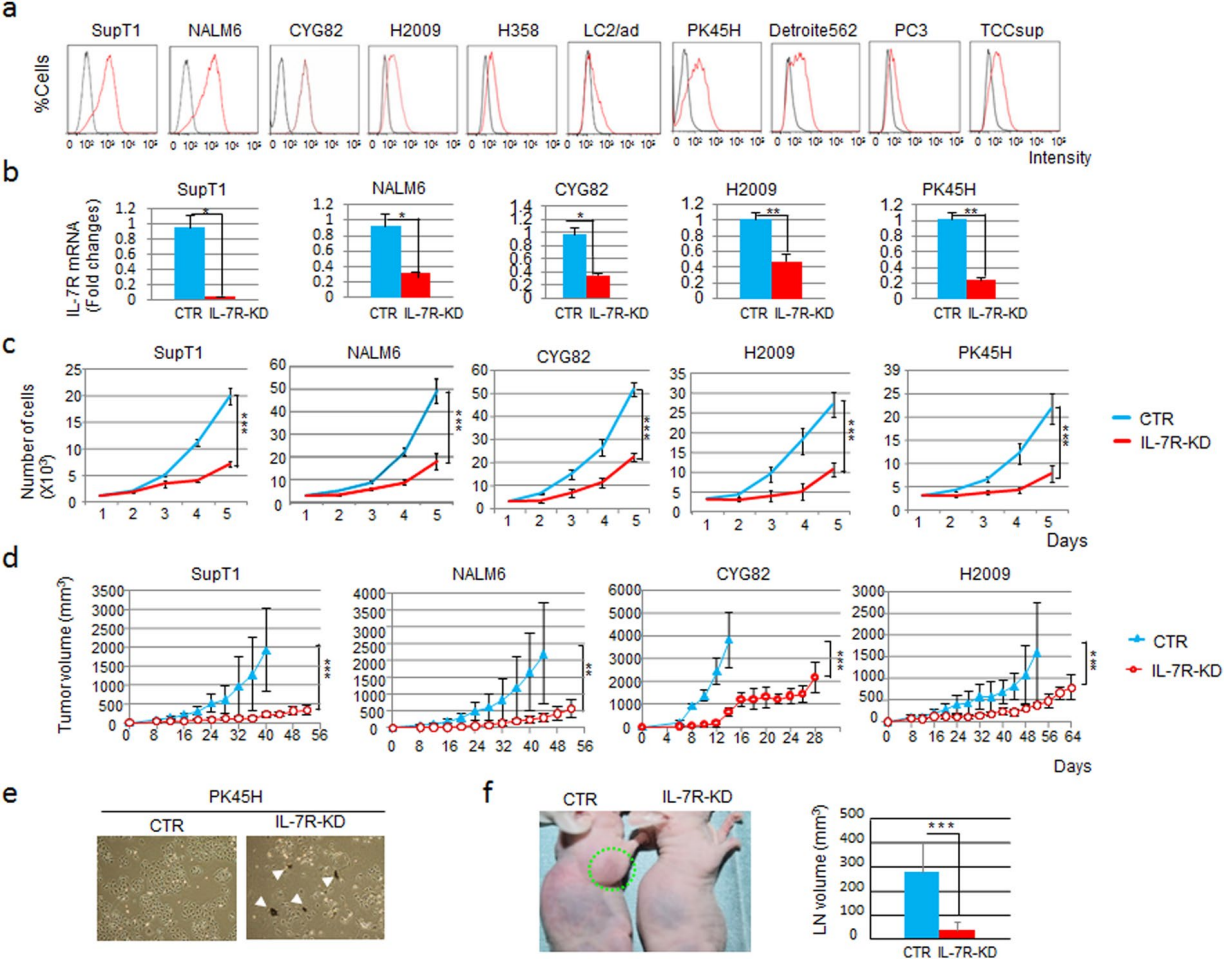
IL-7R knockdown (KD) attenuated cell growth, survival and lymph node (LN) infiltration activity. (**a**) IL-7R expression levels in candidate IL-7R-positive cell lines as analyzed by flow cytometry of antibody-labeled cells. Black, isotype control antibody; red, IL-7R expression. (**b**) Quantitative RT-PCR analysis of IL-7R expression in IL-7R-KD SupT1, NALM6, CYG82, H2009 or PK45H cells compared with the control (CTR) cells (GFP-KD or NS-KD, for the corresponding cell line, as described in materials and methods). NS, non-specific shRNA. Each bar represents the mean ± SD (n = 3). *P < 0.05, **P < 0.01. (**c**) Cell proliferation kinetics of IL-7R-KD and CTR cells. Each bar represents the mean ± SD (n = 3). ***P < 0.001. (**d**) Tumor progression in the xenograft model of IL-7R-KD and CTR cells in mice, as determined by quantification of tumor volume (mm^3^). Each bar represents the mean ± SD (n = 5). **P < 0.01, ***P < 0.001. (**e**) Optical microscopy of IL-7R-KD and CTR PK45H cells. White arrowheads indicate the emergence of dead-cell debris. (**f**) LN infiltration activity of IL-7R-KD and CTR CYG82 cells inoculated into the forelimb palmar pads of mice in a separate group (n = 5) was examined. Massive LN swelling (surrounded by the green dotted line) was observed in mice inoculated with the CTR cells. LN volume was compared between mice inoculated with IL-7R-KD cells and those inoculated with CTR cells. Each bar represents the mean ± SD. ***P < 0.001.

To establish whether IL-7R might be a useful target for treating lymphoid malignancies, we first needed to confirm the involvement of this receptor. For the functional analysis of IL-7R-positive cells, we screened cell lines using a gene expression database (www.oncomine.org). We found that IL-7R was expressed not only malignant lymphoid cells but also in metastatic solid tumor cell lines (Fig. 1a).

To address the biological significance of IL-7R in malignant lymphoid and solid metastatic tumor cells, this gene was knocked down in human T cell lymphoma (SupT1), human B cell leukemia (NALM6), mouse B cell leukemia (CYG82), human metastatic lung cancer (H2009) and human metastatic pancreatic cancer (PK45H) cell lines. IL-7R expression levels were evaluated by quantitative RT-PCR to confirm efficient knockdown (Fig. 1b). Knockdown of IL-7R attenuated both cell growth kinetics *in vitro* (Fig. 1c) and tumor growth in mice (upon subcutaneous injection, for four of the five cell lines; Fig. 1d) relative to control cells. A fifth cell line, PK45H, did not form tumors in mice, and cultured IL-7R-knockdown (IL-7R-KD) PK45H cells showed a substantial proportion of dead cells and cellular debris, which indicated that in these cells, abrogation of IL-7R signaling strongly affected cell survival (Fig. 1e). Furthermore, in a model of LN infiltration in which CYG82 cells were inoculated into the foot pads of mice, significantly decreased LN infiltration by IL-7R-KD CYG82 cells was observed compared to the control cells (Fig. 1f). Thus, IL-7R signaling promotes survival and tumor aggressiveness of both malignant lymphoid and metastatic solid tumor cells.

### IL-7R-dependent steroid-resistance occurs in malignant lymphoid cells

**Figure 2:**
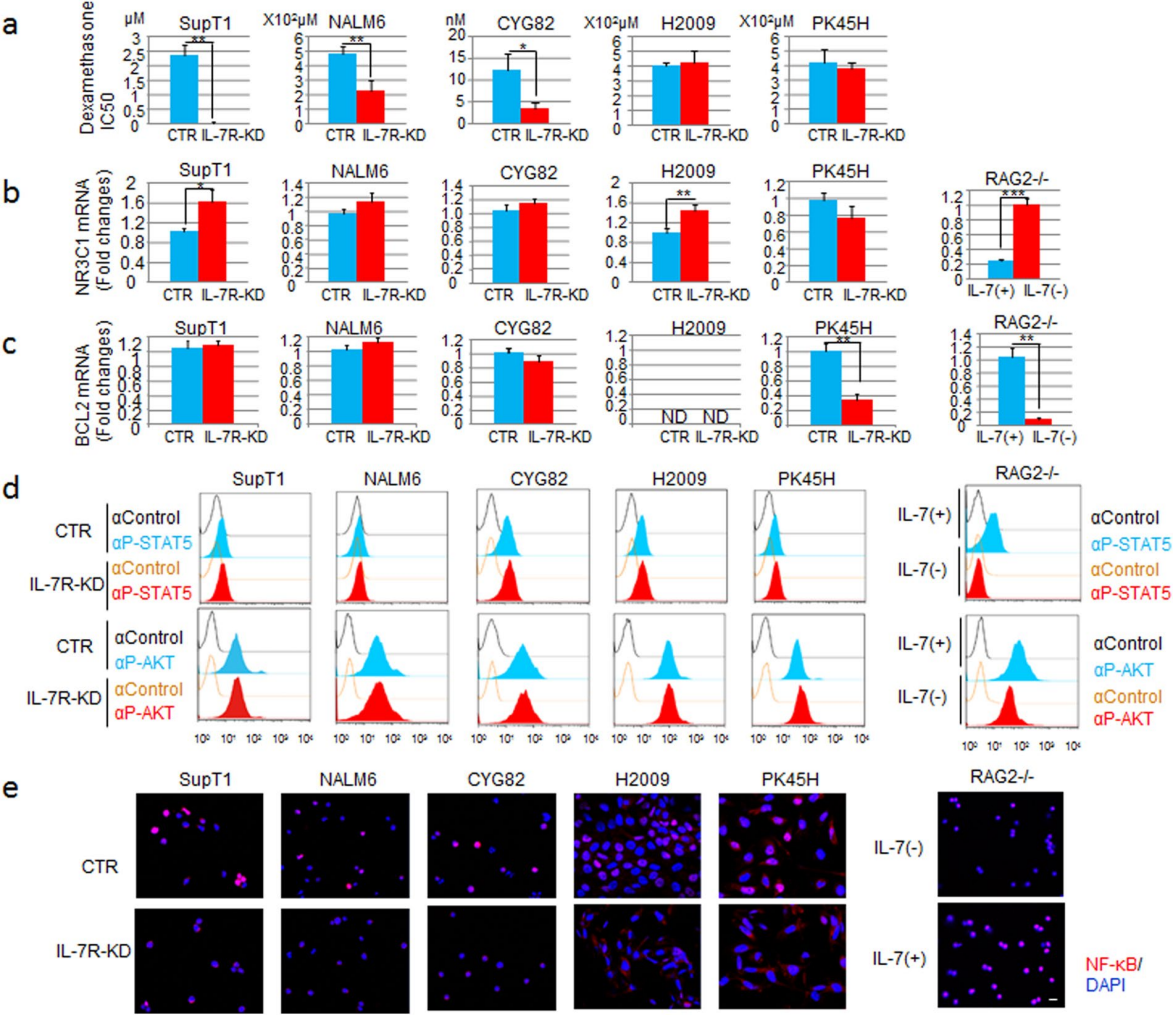
IL-7R KD increased steroid sensitivity in malignant lymphoid cells but not in solid tumor cells. (**a**) IC_50_ values of DEX in IL-7R- KD SupT1, NALM6, CYG82, H2009 or PK45H cells compared with the CTR (GFP-KD or NS-KD) cells. NS, non-specific shRNA; IC_50_, half-maximal inhibitory drug concentration. Each bar represents the mean ± SD (n = 3). *P < 0.05, **P < 0.01. (**b**) NR3C1 expression, as measured by quantitative RT-PCR, was evaluated between IL-7R-KD and CTR cells (left) or between IL-7-dependent RAG2−/− cells with IL-7 (+) and without IL-7 (−) (right). Each bar represents the mean ± SD (n = 3). *P < 0.05, **P < 0.01, ***P < 0.001. (**c**) BCL2 expression, as measured by quantitative RT-PCR, was evaluated between IL-7R-KD and CTR cells (left) or between IL-7-dependent RAG2−/− cells with IL-7 (+) or without IL-7 (−) (right). ND, not detected. Each bar represents the mean ± SD (n = 3). **P < 0.01. (**d**) Comparison of STAT5 (upper) or AKT (lower) phosphorylation levels between IL-7R-KD and CTR cells (left) or between IL-7-dependent RAG2−/− cells with IL-7 (+) or without IL-7 (−) (right) evaluated by flow cytometric analysis of anti-phospho-STAT5 (αP-STAT5)- or anti-phospho-AKT (αP-AKT)-labeled cells. An isotype control antibody (αControl) was used as a negative CTR. (**e**) NF-κB phosphorylation (red) and DAPI (nucleus, blue) immunostaining was compared between IL-7R- KD cells and CTR cells (left) or between IL-7-dependent RAG2−/− cells with IL-7 (+) or without IL-7 (−) (right). Bar, 10 μm.

As steroid-resistance is the main cause of therapeutic resistance in lymphoid malignancies^4–6, 29^, we determined the IC_50_ values of the corticosteroid dexamethasone (DEX) for each of the cell lines and examined how these values were affected by IL-7R knockdown. All three IL-7R-KD malignant lymphoid cell lines showed significantly higher DEX sensitivity than the control cells, though no effect on steroid sensitivity was observed for either of the two metastatic solid tumor cell lines (Fig. 2a).

To investigate the potential mechanisms involved in the observed IL-7R-dependent steroid-resistance, we examined the gene expression levels of the steroid receptor NR3C1 and the anti-apoptotic regulator BCL2. The signal intensity of phospho-STAT5 and phospho-AKT, indicating JAK/STAT and PI3K/AKT pathway activation, respectively, were also evaluated. These pathways have previously been associated with steroid-resistance^5, 6^. Unexpectedly, NR3C1 expression was increased in SupT1 malignant lymphoid cells upon knockdown of IL-7R, but no differences were observed between control and IL-7R-KD NALM6 and CYG82 malignant lymphoid cells (Fig. 2b). IL-7R knockdown did not suppress BCL2 (Fig. 2c), phospho-STAT5 or phospho-AKT (Fig. 2d) expression in these cell lines. Although NR3C1 expression was significantly increased in BCL2-negative H2009 cells upon IL-7R knockdown (Fig. 2b), the sensitivity of these cells to DEX treatment was unchanged (Fig. 2a). Despite the suppression of BCL2 expression upon IL-7R knockdown in PK45H cells (Fig. 2c), there was also no effect on DEX sensitivity. Taken together, these data suggest that the IL-7R-dependent steroid-resistance observed in the malignant lymphoid cell lines was independent of NR3C1, BCL2, JAK/STAT and PI3K/AKT signaling. By contrast, in IL-7-dependent RAG2−/− pro-B cells^30–32^, NR3C1 was up-regulated and other molecules were down-regulated in the absence of IL-7 (Fig. 2b,d). Although these genes were activated physiologically in RAG2−/− cells by the IL-7 ligand binding to IL-7R, they were ligand independent or autoactivated in both malignant lymphoid and metastatic solid tumor cells.

As NF-κB is thought to act downstream of IL-7R, we next analyzed whether this pathway was involved in IL-7R-dependent resistance to DEX treatment by comparing the levels of nuclear phospho-p65/NF-κB between IL-7R-KD and control cells or between RAG2−/− cells with and without IL-7. In all cases, the levels of nuclear phospho-p65/NF-κB were positively correlated with IL-7R signaling activation (Fig. 2e). In IL-7R-KD metastatic solid tumor cells in particular, the level of cytoplasmic phospho-p65/NF-κB was substantially increased, thus indicating NF-κB pathway suppression (Fig. 2e). Nevertheless, DEX sensitivity was not affected in these IL-7R-KD cells. These results suggest that NF-κB activation downstream of IL-7R signaling might promote tumor growth rather than DEX resistance. Overall, our data suggest that IL-7R signaling contributes to steroid-resistance independent of NR3C1, BCL2, JAK/STAT, PI3K/AKT or NF-κB.

### Preparation and characterization of an anti-IL-7R antibody-drug conjugate (A7R-ADC)

**Figure 3:**
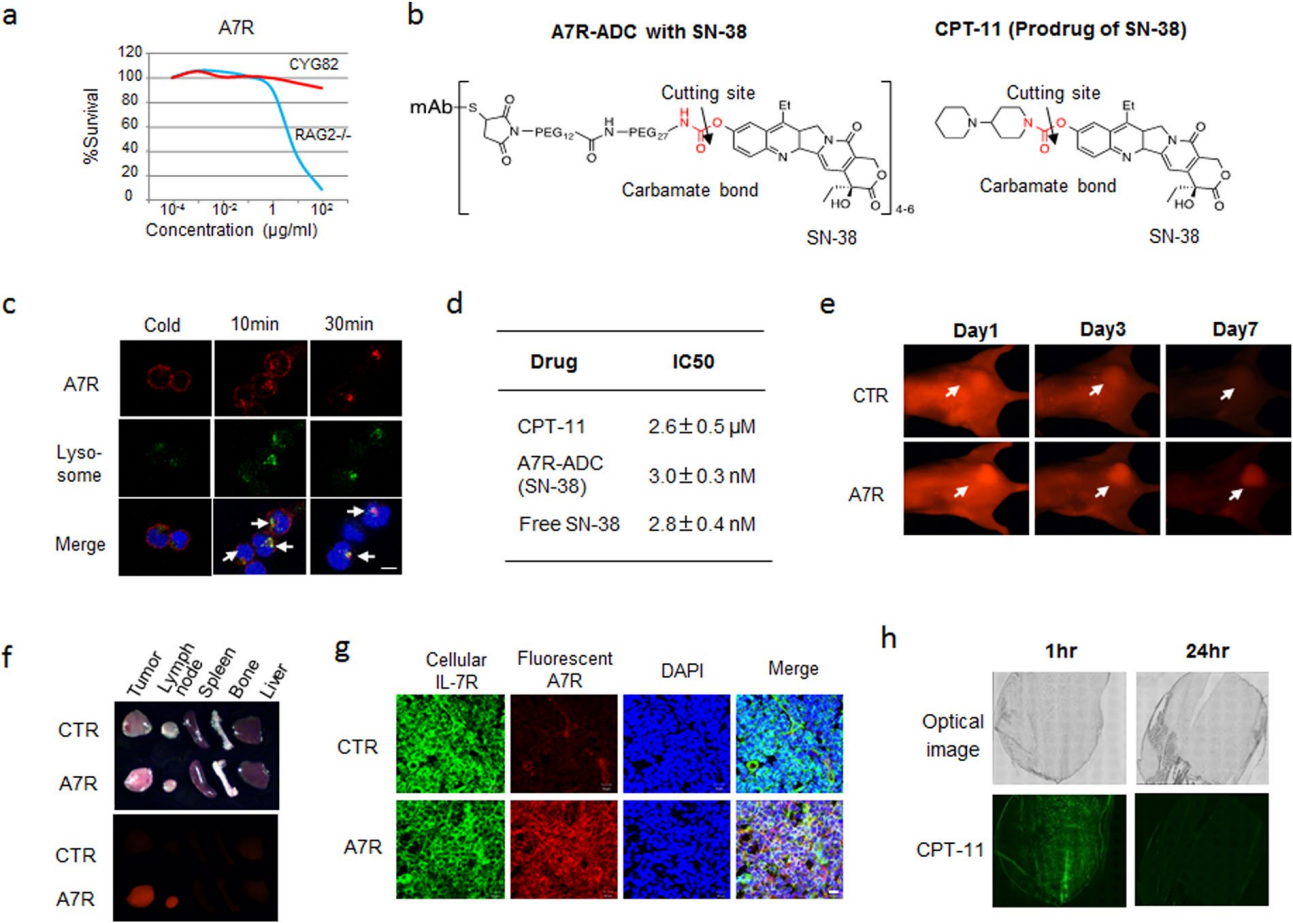
Preparation and characterization of A7R-ADC-SN-38. (**a**) *In vitro* cytotoxic effect of A7R against RAG2−/− or CYG82 cells. (**b**)Drug design of A7R-ADC-SN-38. SN-38 was conjugated to A7R via a carbamate bond (red). CPT-11, as a prodrug of SN-38, was used as a CTR with a carbamate bond (red). Arrows indicate the cleavage site. (**c**)Internalization of fluorescent A7R (red) into CYG82 cells was examined at 10 or 30 minutes (min) after the incubation. A low temperature (cold) incubation at 4 °C, in which the internalization was blocked, was conducted as a CTR. The arrows show merged yellow as co-localization with lysosome (green). Scale bar, 5 μm. (**d**) *In vitro* cytotoxic effect of A7R-ADC-SN-38 in CYG82 cells was compared with that of CPT-11 or free SN-38. (**e**) *In vivo* imaging analysis of CYG82 tumors was conducted using fluorescent A7R on days 1, 3 and 7 after injection. The arrows indicate each tumor position. A fluorescent isotype control antibody was used as a CTR. (**f**) *Ex vivo* imaging of A7R delivery into the tumors, lymph nodes, spleens, bones and livers in mice with CYG82 tumors. (**g**) The intra-CYG82 tumor distribution of fluorescent A7R (red) examined at 24 hours after injection. Immunohistochemistry with cellular IL-7R (green) and DAPI (nucleus, blue). Yellow indicates the overlap of the injected A7R and IL-7R in the tumor cells. A fluorescent isotype control antibody was used as a CTR. Scale bar, 10 μm. (**h**) The intra-CYG82 tumor distribution of CPT-11 (fluorescence of CPT-11, green).

As IL-7R expression and signaling influenced tumor growth and resistance to steroids, we hypothesized that a therapy targeting IL-7R might be effective against such refractory malignant cells. First, we evaluated the cytotoxic activity of an anti-mouse IL-7R neutralizing antibody (clone A7R34; A7R)^12, 14^ against IL-7-independent mouse leukemia CYG82 cells. A7R showed a significant cytotoxic effect against IL-7-dependent pro-B cell RAG2−/− cells, but not CYG82 cells (Fig. 3a). Next, we used ADC technology to determine whether using A7R to deliver a cytotoxic agent, or payload, to target cells could result in a more selective and efficient elimination of these cells than that achieved by monotherapy with mAb or ACA administration alone. SN-38—an active form of the prodrug CPT-11, which is used to treat many types of cancers (including lymphoid malignancies^33, 34^)—was selected as the payload for A7R-ADC development. We also developed anti-CD20-ADC connected via specialized linker with a carbamate bond, for use against lymphoid malignancy; we confirmed that the ADC was stable in the blood and that good blood stability and *in vitro* efficient release activity of the linker^34^. Therefore, SN-38 was conjugated to A7R via the same carbamate bonding in generating A7R-ADC-SN-38 (Fig. 3b). First, we determined whether A7R, though not cytotoxic *per se*, would act as an effective payload delivery method in the form of A7R-ADC-SN-38. Upon incubation with CYG82 cells, A7R was internalized efficiently and accumulated within lysosomes (Fig. 3c). According to our observations, A7R-ADC-SN-38 showed the same strong cytotoxicity as free SN-38 (Fig. 3d). *In vivo* and *ex vivo* imaging showed A7R accumulation in both primary CYG82 tumor cells and secondary infiltrated LNs in mice (Fig. 3e and f), which was still observable 7 days after treatment in the case of primary CYG82 tumors (Fig. 3e). A7R was distributed throughout the entire tumor area, and the ADCs bound to and were internalized by IL-7R-positive CYG82 cells (Fig. 3g). We also examined the distribution of CPT-11 in the same tumor model. Although strong accumulation was observed at 1 hour after injection, this accumulation rapidly cleared within 24 hours (Fig. 3h), indicating that A7R, and thus presumably also A7R-ADC-SN-38, persists longer than CPT-11 in CYG82 tumors. These data demonstrate that A7R exhibits favorable properties in terms of internalization, accumulation and duration within cells and tumors to deliver drugs to target cells. In line with this hypothesis, A7R-ADC-SN-38 exhibited a cytotoxic effect that was stronger than that of CPT-11 and equal to that of free SN-38 upon incubation with CYG82 cells *in vitro* (Fig. 3d). Thus, with the use of ADC technology, A7R, though not cytotoxic *per se*, can deliver cytotoxic agents to efficiently eliminate tumor cells.

### A7R-ADC-SN-38 suppresses primary tumor growth and secondary LN infiltration

**Figure 4:**
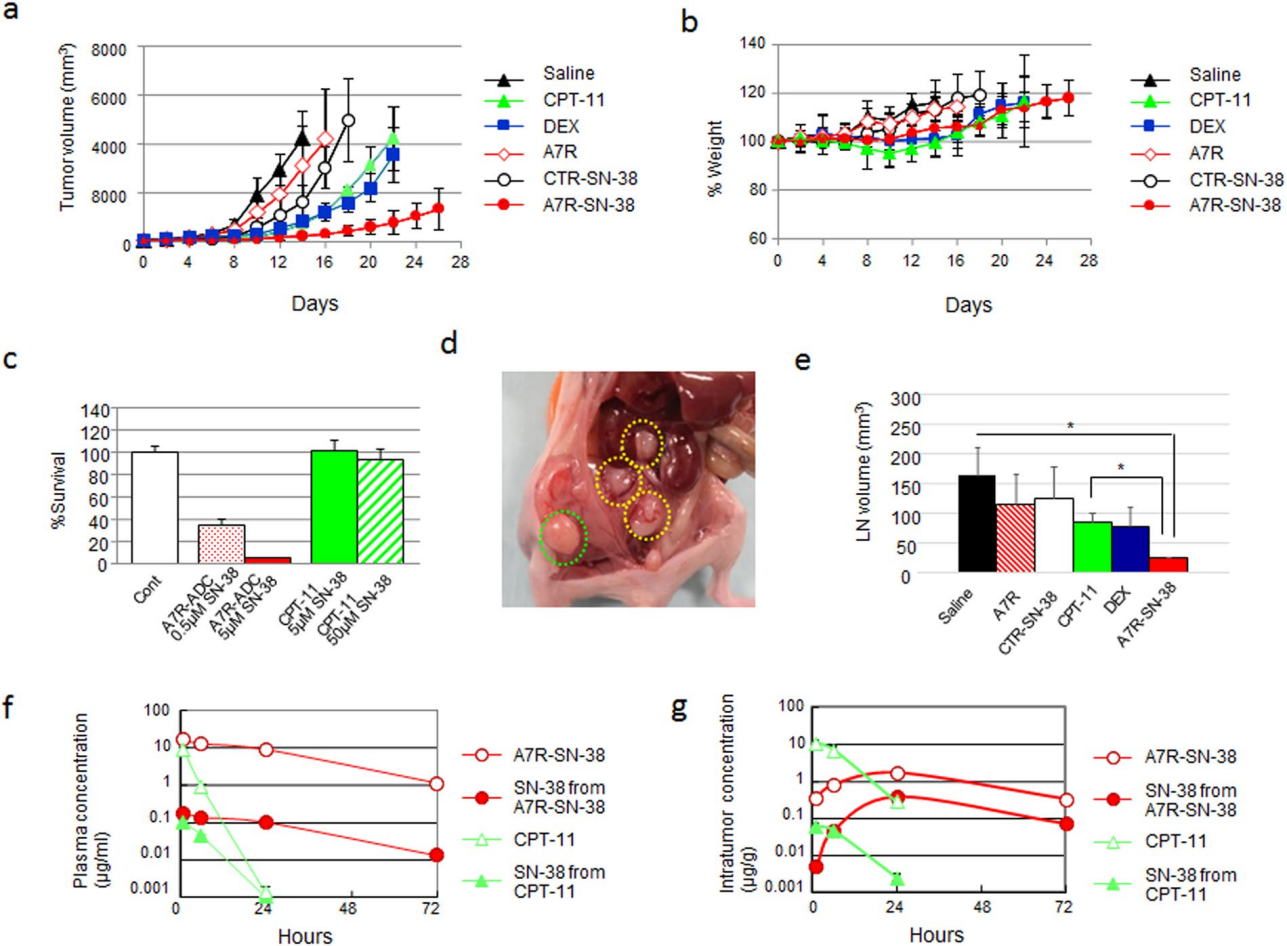
Anti-tumor effect of A7R-ADC-SN-38 on the CYG82 tumor model. (**a**) Anti-tumor activities were examined. In animal models of CYG82 cells, an isotype control ADC with SN-38 (CTR-SN-38) or A7R with SN-38 (A7R-SN-38) was administered at a dose equivalent to 0.6 mg/kg of SN-38 to separate groups of mice (n = 5) through intravenous bolus injections on days 0, 4, and 8. CPT-11 at a dose equivalent to 11.6 mg/kg of SN-38, A7R at an equivalent antibody dose of 50 mg/kg, high-dose DEX at 10 mg/kg and saline as a control were also administered on the same schedule as ADCs. The curves illustrate the effects of the treatments on tumor size. P < 0.05 (CTR-SN-38 vs. A7R). P < 0.001 (saline vs. CTR-SN-38, CPT-11, DEX or A7R-SN-38; A7R-SN-38 vs. A7R, CTR-SN-38, CPT-11 or DEX; DEX vs. A7R or CTR-SN-38; CPT-11 vs. A7R or CTR-SN-38). Bar = SD. (**b**) The % change in body weight was examined in the same mice (n = 5) with the same treatment shown in (A). Bar = SD. (**c**) *In vitro* cytotoxic effects of A7R-ADC or CPT-11 were examined 72 hours after a short incubation treatment (1 hour). The doses were administered as equivalent amounts to SN-38 doses for the comparison. P < 0.001 (A7R-ADC [0.5 μM SN-38] vs. CPT-11 [5 μM SN-38] or CPT-11 [50 μM SN-38], or A7R-ADC [5 μM SN-38] vs. CPT-11 [5 μM SN-38] or CPT-11 [50 μM SN-38]). Each bar represents the mean ± SD (n = 6). (**d**) CYG82 xenograft model. Inguinal LNs (surrounded by the green dotted line) and intra-abdominal LNs (surrounded by the yellow dotted lines) were observed. (**e**) Inhibition of LN infiltration was examined in a separate group of mice (n = 3) with the same treatment shown in (A). Each bar represents the mean ± SD. *P < 0.05. (**f** and **g**). Plasma concentrations (**f**) or tumor concentrations (**g**) of total (bound and unbound) SN-38 and free (unbound) SN-38 from CPT-11 or A7R-SN-38 were determined using HPLC.

Given that A7R-ADC-SN-38 exhibited cytotoxic activity *in vitro*, the anti-tumor activity of A7R-ADC-SN-38 was evaluated *in vivo* using a syngeneic mouse model. To do this, we compared its therapeutic potential with that of steroid treatment, the neutralizing A7R antibody alone, a control ADC-SN-38 conjugate or the prodrug CPT-11. Although treatment with A7R (50 mg/kg) alone did not have a significant effect, CYG82 tumor growth was significantly suppressed after treatment with DEX (10 mg/kg), CPT-11 (20 mg/kg/day; equivalent to an 11.6 mg/kg dose of SN-38), an isotype control ADC (CTR-ADC, 50 mg/kg) with SN-38 or A7R-ADC-SN-38 (50 mg/kg; equivalent to a 0.6 mg/kg dose of SN-38 in both ADCs) compared with saline. Of these treatments, A7R-ADC-SN-38 showed the strongest and most significant anti-tumor effect. Tumor volumes were 779.4 mm^3^, 3542.5 mm^3^ and 4216.8 mm^3^ at day 22 after the administration of A7R-ADC-SN-38, DEX and CPT-11, respectively (Fig. 4a). Regarding toxicity, there was no clear body weight loss in the mice treated with A7R-ADC-SN-38 and DEX, whereas CPT-11-treated mice showed a 5% decrease in body weight during the treatment (Fig. 4b).

We next evaluated the differences in cytotoxic effects between A7R-ADC-SN-38 and CPT-11 after a short incubation period (1 hour) to mimic the reaction between drugs and malignant cells in the blood stream. A7R-ADC-SN-38 showed a stronger cytotoxic effect than CPT-11 (Fig. 4c), suggesting that this ADC would also be effective against circulating IL-7R-positive tumor cells. In a CYG82 tumor model, abdominal aggressive LN infiltration from the primary site was observed (Fig. 4d). No significant decrease in LN size was seen upon treatment with any of the agents except A7R-ADC-SN-38, when compared to the saline control, which effectively reduced LN volume 0.15-fold (Fig. 4e). In a pharmacokinetic analysis of A7R-ADC-SN-38, total SN-38 (A7R-bound form and unbound form) and free SN-38 (unbound form) in plasma declined gradually by 72 hours, whereas CPT-11 showed rapid clearance within 24 hours (Fig. 4f). A high concentration of both total and free SN-38 in tumor tissue after treatment with A7R-ADC-SN-38 was observed compared with CPT-11 (Fig. 4g). Collectively, these results demonstrate that A7R-ADC-SN-38 is a more potent anti-tumor drug than the SN-38 prodrug CPT-11.

### A7R-ADC-SN-38 is effective against steroid-resistant CYG82 cells

**Figure 5:**
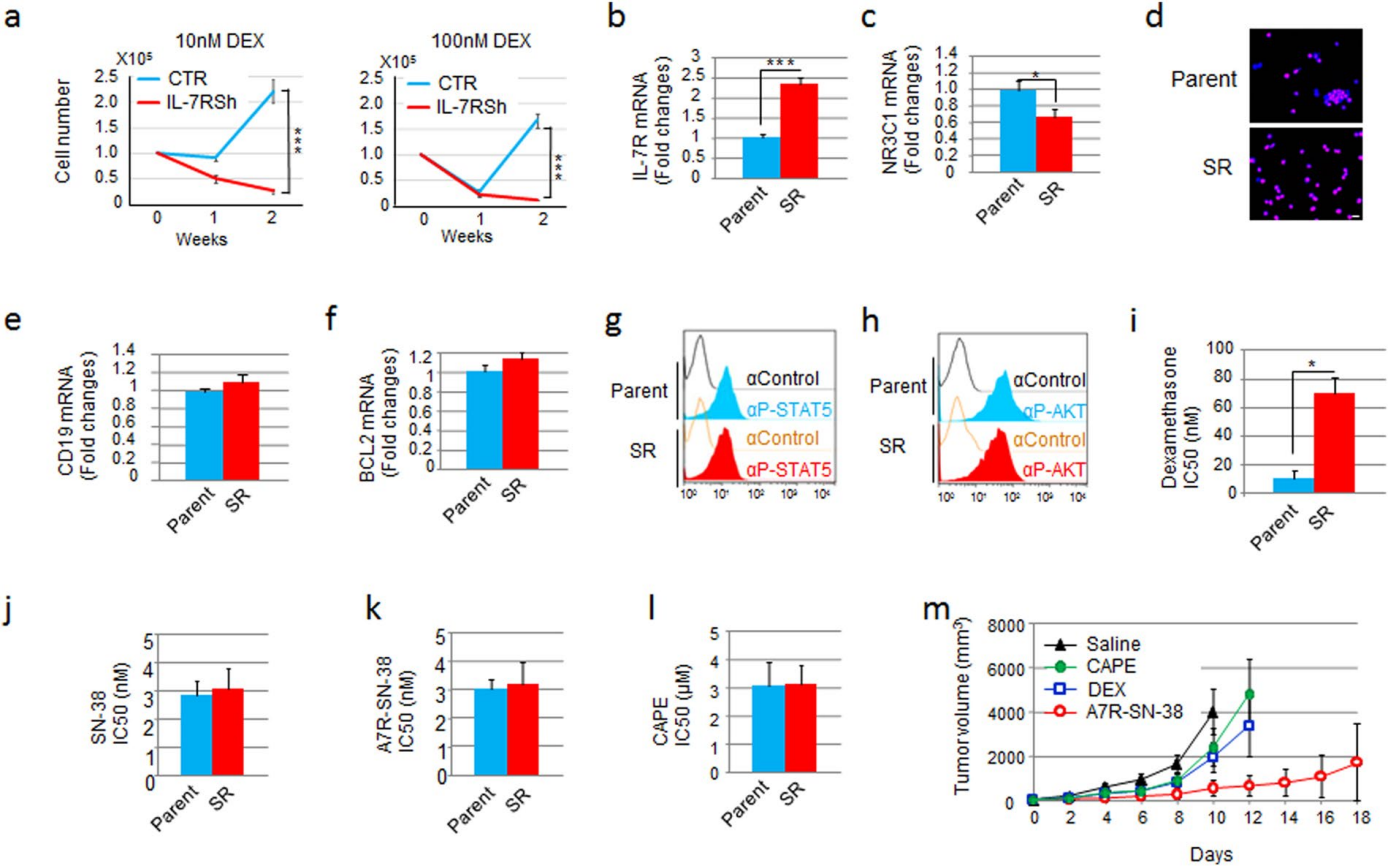
A7R-ADC-SN-38 is effective against the IL-7R-up-regulated CYG82 tumor model with steroid-resistance. (**a**) Cell growth kinetics were examined in IL-7R-KD and control (CTR) cells incubated with high-dose (10 or 100 nM) DEX for 2 weeks. Each bar represents n = 3; mean ± SD. ***P < 0.001. (**b** and **c**). Quantitative RT-PCR results of IL-7R (**b**) or NR3C1 (**c**) expression were evaluated between parent and steroid-resistant (SR) CYG82 cells. Each bar represents n = 3; mean ± SD. *P < 0.05, ***P < 0.001. (**d**) NF-κB (red) and DAPI (nucleus, blue) immunostaining was compared between parent and SR CYG82 cells. Scale bar, 10 μm. (**e** and **f**). CD19 (**e**) or BCL2 (**f**) expression, as measured by quantitative RT-PCR, was evaluated between parent and SR CYG82 cells. Each bar represents the mean ± SD (n = 3). (**g** and **h**) g and h. STAT5 (**g**) or AKT (**h**) phosphorylation was evaluated between parent and SR CYG82 cells using flow cytometry with an anti-phospho-STAT5 (αP-STAT5) or anti-phospho-AKT (αP-AKT) antibody. (**i**–**l**) i–l. IC_50_ values of DEX (**i**), SN-38 (**j**), A7R-ADC-SN-38 (**k**) or the NF-κB inhibitor CAPE (**l**) of parent and SR CYG82 cells were examined. Each bar represents the mean ± SD (n = 3). *P < 0.05 (**m**) Anti-tumor activities were examined in animals injected with SR CYG82 cells, with saline as a control. The NF-κB inhibitor CAPE at 20 mg/kg, DEX at 10 mg/kg or A7R-SN-38 at 0.6 mg/kg as equivalent to a dose of SN-38 was administered to the mice (n = 5) through intravenous bolus injections on days 0, 4, and 8. The curves illustrate the effects of the treatments on tumor size. P < 0.01 (saline vs. DEX), P < 0.001 (A7R-SN-38 vs. saline, CAPE or DEX). Bar = SD.

As steroid-resistance is a major problem in the treatment of malignant lymphoid cells^4, 5, 29^, and we observed that CYG82 cells readily acquired steroid-resistance when treated with DEX, we next examined whether the acquisition of steroid-resistance was dependent on IL-7R signaling. Control CYG82 cells, but not IL-7R-KD cells, survived and exhibited regrowth under high-dose DEX treatment (Fig. 5a). We next compared the characteristics between parent and steroid-resistant (SR) CYG82 cells. SR CYG82 cells showed significantly higher IL-7R expression and significantly lower NR3C1 expression than parent CYG82 cells (Fig. 5b and c). Moreover, most SR CYG82 cells were positive for nuclear phospho-p65/NF-κB staining (Fig. 5d). By contrast, there were no differences in CD19 or BCL2 expression and phospho-STAT5 or phospho-AKT intensity between parent and SR CYG82 cells (Fig. 5e–h). Although DEX became less effective against SR CYG82 cells, SN-38, A7R-ADC-SN-38 and the NF-κB inhibitor caffeic acid phenethyl ester (CAPE)^35^, were all effective against both parent and SR CYG82 cells *in vitro* (Fig. 5i–l). We next evaluated the anti-tumor effect of DEX (10 mg/kg), CAPE (20 mg/kg) and A7R-ADC-SN-38 (50 mg/kg; equivalent to a 0.6 mg/kg dose of SN-38) in SR CYG82 tumors. DEX treatment exhibited a weak but significant ability to reduce the SR CYG82 tumor volume compared to saline, though CAPE treatment had no effect (Fig. 5m). By contrast, A7R-ADC-SN-38 treatment had a significantly stronger anti-tumor effect than all other treatments, resulting in a tumor volume 0.18-fold lower than in mice treated with DEX at day 12 (Fig. 5m). Thus, A7R-ADC-SN-38 showed potent anti-tumor effects against steroid-resistant lymphoid malignancy.

We also examined the relationship between IL-7R expression and steroid-resistance in clinical samples by using data from a publicly available database (www.ncbi.nlm.nih.gov/geo/query/acc.cgi?acc=GSE39339, GSE32962, or http://www.embl-ebi.ac.uk/arrayexpress/experiments/E-MEXP-3916). In the analysis of GSE39339, GSE32962 and E-MEXP-3916, IL-7R expression associated with steroid treatment or resistance was significantly increased or sustained relative to that of B- or T-cell markers including CD19, CD22 or CD3e (Supplementary Fig. 1). Collectively, these data suggest that enhanced IL-7R expression may be a good indicator of steroid-resistant lymphoid malignancies and that A7R-ADC might provide an effective means to treat such tumors *in vivo*.

### Physiological and pathological steroid-resistant IL-7R-positive lymphocytes can be eliminated by A7R-ADC

**Figure 6:**
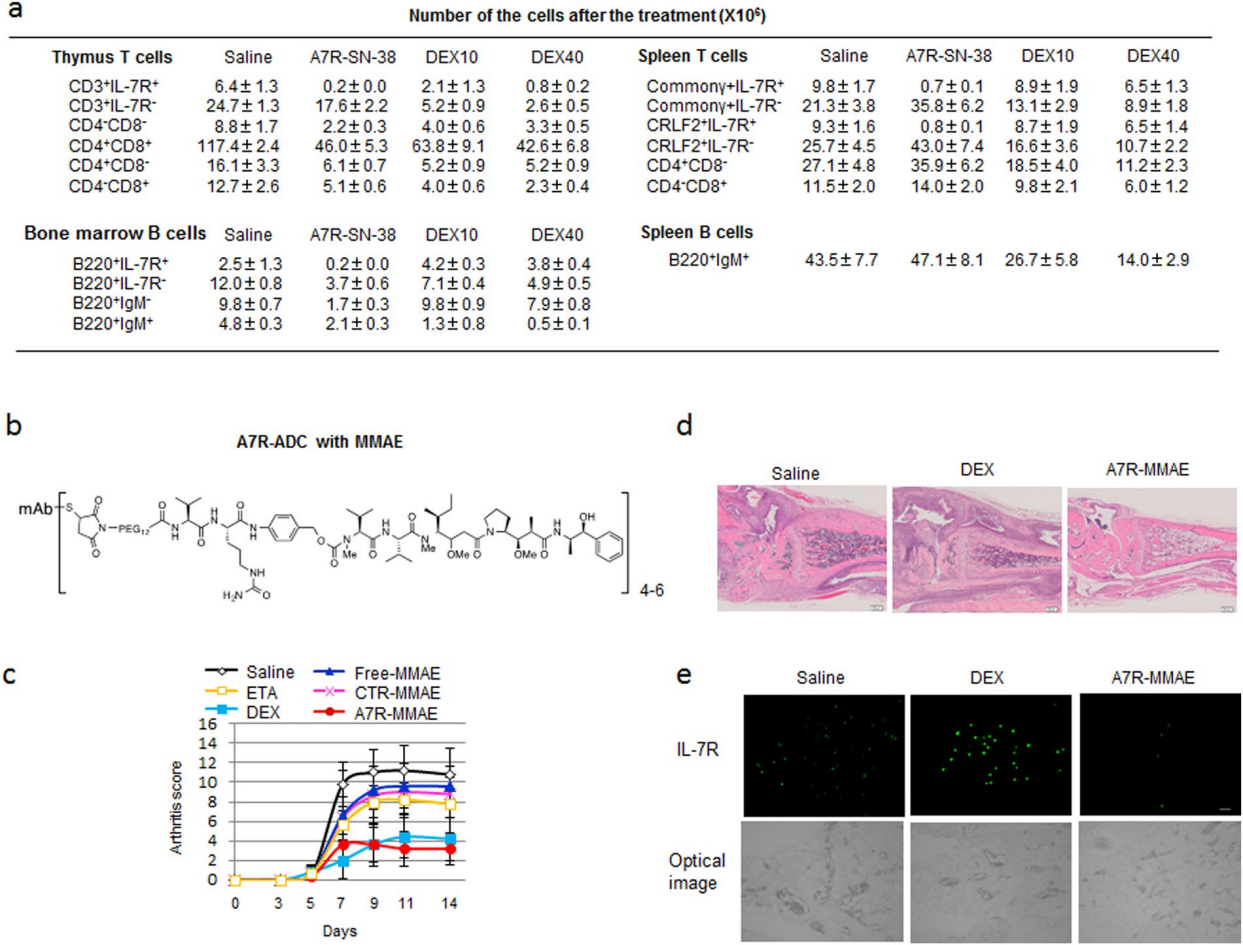
A7R-ADC eliminates steroid-resistant IL-7R-positive lymphocytes in physiological bone marrow and spleen or autoimmune arthritis. (**a**) Changes in cell numbers in response to A7R-ADC-SN-38 treatment (0.6 mg/kg as an equivalent SN-38 dose) or high-dose DEX (10 or 40 mg/kg) at 7 days after injection. Each bar represents the mean ± SD (n = 3). (**b**) Drug design of anti-IL-7R ADC with MMAE. MMAE was conjugated to an anti-IL-7R monoclonal antibody (A7R) via a Val-Cit linker. (**c**) Kinetics of arthritis scores in mice with collagen antibody-induced arthritis (CAIA) were evaluated using saline as a control, the TNF inhibitor etanercept (ETA, 30 mg/kg), DEX (10 mg/kg), free MMAE (0.6 mg/kg), control ADC with MMAE (CTR-MMAE, 30 mg/kg) or A7R-ADC-MMAE (A7R-MMAE, 30 mg/kg, both ADCs; 0.6 mg/kg as an equivalent MMAE dose) (n = 5). P < 0.01 (saline vs. ETA; A7R-MMAE vs. CTR-MMAE; DEX vs. CTR-MMAE), P <  0.001 (saline vs. DEX or A7R-MMAE; A7R-MMAE vs. ETA or free-MMAE; DEX vs. ETA or free-MMAE). Bar = SD. (**d**) Comparison of joint histology in mice with CAIA treated with saline, DEX and A7R-MMAE. H.E., hematoxylin and eosin staining. (**e**) Immunohistochemical staining of IL-7R in the inflammation site of CAIA compared with that in mice treated with saline, DEX and A7R-MMAE. Top, IL-7R-positive cells (green); bottom, optical image.

Although low expression of IL-7R was detected in testis and female gonad without lymphoid cells (i.e., healthy tissues) in the analysis of a publicly available database (http://pax-db.org/), expression was much lower than that of HER2 or EGFR (Supplementary Table 1). Therefore, we predicted that the frequency of harmful events caused by A7R-ADC would be low in comparison to anti-HER2 or EGFR-ADC. On the other hand, It has also been reported that congenital mutation of the human IL-7R gene causes severe immunodeficiency^36^. Therefore, immunosuppression is a major issue that may limit the clinical application of IL-7R-ADC. We thus evaluated the immunosuppressive effect of A7R-ADC-SN-38 capable of targeting IL-7R-positive normal mouse cells *in vivo* as compared with DEX (10 or 40 mg/kg as the maximum tolerated dose). Although IL-7R-positive cells were substantially eliminated by A7R-ADC-SN-38 treatment, B220-positive IgM-positive mature B cells and single CD4-positive or CD8-positive T cells were conserved. We also examined the expression of the common γ chain, a critical subunit that is common to the receptor complexes for the interleukin receptors of IL-2, IL-4, IL-7, IL-9, IL-15 and IL-21, and CRLF2, which forms a heterodimer receptor with IL-7R for TSLP. As the number of cells that were negative for IL-7R and positive for the common γ chain or CRLF2 increased, we concluded that other IL-7R-independent immune-related cytokine signals were left intact by A7R-ADC-SN-38 treatment. By contrast, we observed that the number of IL-7R-positive bone marrow B cells increased 1.5–1.7-fold in response to treatment with high-dose DEX. In addition, although IL-7R-positive thymus T cells showed a 0.1- or 0.3-fold decrease, the majority (66–93%) of IL-7R-positive splenic T cells survived under the same treatment. Conversely, treatment with A7R-ADC-SN-38 suppressed all IL-7R-positive B and T lymphocytes in the thymus, bone marrow and spleen (Fig. 6a, Supplementary Fig. 2).

As A7R-ADC treatment appeared to eliminate IL-7R-positive cells strongly and specifically, we hypothesized that A7R-ADC might have distinctive immunosuppressive activity that could be effective against various autoimmune diseases, particularly in steroid-resistant conditions. We chose to focus on rheumatoid arthritis (RA), as IL-7R-positive lymphocytes have been implicated in the pathogenesis of this disease^26, 37, 38^, and verified that IL-7R was highly expressed in the inflammation sites of RA patients by analyzing clinical data from a public database (www.ncbi.nlm.nih.gov/geo/query/acc.cgi?acc=GSE1919, GSE55457, Supplementary Fig. 3). We then examined the effectiveness of A7R-ADC-SN-38 compared with A7R or DEX treatment. SN-38 conjugated with an anti-CD19 antibody (anti-CD19-ADC-SN-38) was also used as a control. A7R-ADC-SN-38 showed a significant anti-inflammatory effect and decreased arthritis score of 6.0 relative to the scores of 11.0, 9.8, and 8.5 obtained after treatment with saline, A7R and ACD19-ADC-SN-38, respectively (Supplementary Fig. 4).

Although the anti-inflammatory effect of A7R-ADC-SN-38 was weaker than that of DEX treatment, we observed that the forelimbs of some DEX-treated mice showed strong inflammation, representing sites of uncontrolled inflammation. Therefore, we hypothesized that steroid-resistant IL-7R-positive cells may maintain inflammation at these sites. To target these remaining cells, we developed an alternative IL-7R-targeting ADC for the treatment of steroid-resistant arthritis using monomethyl auristatin E (MMAE)^39, 40^ as a payload due to its more potent cytotoxicity compared to that of SN-38. MMAE was conjugated to A7R (A7R-ADC-MMAE) via a valine-citrulline linker^39, 40^ (Fig. 6b). As expected, A7R-ADC-MMAE treatment resulted in a decrease in the arthritis score to 3.2 compared to the score of 4.2 observed after DEX treatment, but the difference was not significant (Fig. 6c). More importantly, A7R-ADC-MMAE significantly suppressed inflammation in both the fore and hind legs (Fig. 6d), unlike A7R-ADC-SN-38. Immunohistochemical staining revealed a clear increase in IL-7R^high^-positive cells at uncontrolled inflammation sites in mice treated with DEX compared to those treated with saline. However, only a few IL-7R-positive cells were observed in mice treated with A7R-ADC-MMAE (Fig. 6e), demonstrating that A7R-ADC effectively counteracted both steroid-sensitive and steroid-resistant cell types in this model of arthritis.

## Discussion

The present study indicated that unique IL-7R signaling causes steroid-resistance in lymphoid malignancy. Moreover, it was suggested that ligand independency or autoactivation of downstream molecules abrogates the neutralizing effect of A7R monotherapy, which is a potential drawback to the mAb approach. We also observed the presence of steroid-resistant IL-7R-positive cells physiologically in mouse bone marrow and spleen or pathologically in the inflammation site of a mouse model of autoimmune arthritis. To overcome this steroid-resistance, we have developed A7R-ADC for the treatment of these diseases. Delivered and released potent cytotoxic agents could efficiently eliminate the IL-7R-positive steroid-resistant malignant lymphoid or autoimmune-reactive cells.

In the first part of this study, we observed that IL-7R expression in both malignant lymphoid cells and metastatic solid tumor cells increased cell growth and survival activity and was associated with tumor aggressiveness. Interestingly, a marked difference was observed between these cell types, in that IL-7R signaling influenced steroid sensitivity only in malignant lymphoid cells and not in metastatic solid tumor cells. Although nuclear phospho-p63/NF-κB was inhibited in all IL-7R-KD cells, we considered the NF-κB pathway to be associated with tumor growth rather than steroid-resistance. On the other hand, the association of NR3C1, BCL2, JAK/STAT and PI3K/AKT with steroid-resistance^5, 6^ did not sufficiently explain the difference between these cell types.

Previously, several authors reported high expression of IL-7R on tumor cells^18, 41, 42^. Among them, Nakase indicated that IL-7R was widely expressed with more than 2000 sites/cell, similar to other cytokine receptor such as IL-3R, IL-2R, KIT, and FLT3^41^. In this study, an as yet uncharacterized pathway downstream of IL-7R signaling may contribute to steroid-resistance specific to lymphoid malignancies. Indeed, IL-7R signaling was necessary for CYG82 cells to acquire steroid-resistance, and steroid-resistant CYG82 cells showed higher IL-7R expression than did parental CYG82 cells. An analysis of clinical data^29, 43, 44^ further supported this association between IL-7R and steroid-resistance, with increased IL-7R expression in cells correlating with either steroid treatment or steroid-resistance. Furthermore, the expression levels of CD19, CD22 and CD3e, standard markers of lymphoid malignancies, were decreased or unchanged by steroid treatment or resistance (Supplementary Fig. 1). Clinical trials with anti-CD19-ADC-monomethyl auristatin F (MMAF) and anti-CD22-ADC-calicheamicin are currently underway. They are expected to be used to treat relapsed B lymphoid malignancy that occurs after the cyclophosphamide, vincristine, adriamycin and dexamethasone (hyper-CVAD) regimen or as a first-line treatment in combination with hyper-CVAD^45^. Although an anti-CD3e antibody has been used as a bispecific antibody to activate cytotoxic T cells, very few ADCs have been developed for the treatment of T lymphoid malignancy^46, 47^. In any case, it is necessary to overcome steroid-resistance to elevate the response rate. Therefore, in these clinical situations, IL-7R targeting may be more advantageous than that of CD19, CD22 or CD3e.

Potential drawbacks to the clinical application of A7R-ADC therapy include off-target toxicities and severe immunosuppression^36^. However, regarding the former possibility, we confirmed that the numbers of moderately and highly IL-7R-positive lymphoid cells were limited. Moreover, A7R-ADC did not cause significant weight loss in a preclinical setting. Regarding the latter possibility, we confirmed that in mature B220-positive IgM-positive B cells and single CD4-positive or CD8-positive T cells survived IL-7R-ADC treatment in mice. Moreover, IL-7R knockout mice are healthy, fertile and immune responsive in a normal breeding environment^13, 14, 48, 49^. These observations imply that the host defense response is maintained even after the complete elimination of IL-7R-positive cells, minimizing the infection risk. Therefore, over a limited period of time, immunosuppression by A7R-ADC would be well tolerated clinically. Recently, it has been reported that IL-7R–positive innate lymphoid cells (ILCs) play significant roles in host immunity and inflammation, especially in gut mucosal homeostasis^50–53^. Although significant digestive disorders such as diarrhea or bloody stool were not observed in this study, further investigation is needed to elucidate the influence of A7R-ADC on ILCs in other preclinical settings, including some immune or inflammatory disease models.

Homeostatic proliferation of T lymphocytes is regulated by two independent processes, thymic function and postthymic peripheral function. Steroid treatment suppresses the former more strongly than the latter^54^. Spleen B lymphocytes are also more susceptible to steroid-induced apoptosis compared with bone marrow B lymphocytes^55^. Thus, although the main biological activity of steroids is the suppression of T and B lymphopoiesis, heterogeneous subpopulation-specific reactions can be observed. In the present study, we also observed that IL-7R-positive bone marrow B cells and splenic T cells survived under high-dose steroid treatment *in vivo*. Moreover, IL-7R-positive lymphocytes have been implicated in the pathogenesis of autoimmune diseases^8, 22–24, 26, 37, 38^, and these cells survived and maintained inflammation in our mouse arthritis model under steroid treatment. Steroid-induced up-regulation of IL-7R expression has been shown to contribute to enhanced survival and function of T cells *in vitro* ^56, 57^. Therefore, the potent steroid-resistance of IL-7R-positive cells might be a common feature of both normal conditions and the pathological conditions of lymphoid malignancy and autoimmune disease. This suggests that targeting these cells through A7R-ADC treatment might also provide a novel therapeutic approach in such situations. Interestingly, it has been demonstrated that patients with autoimmune diseases have an increased risk of developing lymphoid malignancies^58^. Steroid-induced enhancement of IL-7R signaling may contribute to this pathogenesis. Conversely, the development of autoimmune diseases can be observed in some patients following immune checkpoint blockade^2^. Strong and specific elimination of enhanced IL-7R-positive cells, as a common pathogenesis of both lymphoid malignancy and autoimmune disease, might prevent the development of malignancy or autoimmune disease in high-risk patients. Therefore, A7R-ADC may be useful in the treatment of these patients.

A7R-ADC-SN-38 had a stronger anti-inflammatory effect than A7R monotherapy or anti-CD19 ADC with SN-38 treatment in the mouse autoimmune arthritis model. However, this effect was insufficient to suppress the observed inflammation. Clinically, MMAE exhibited much stronger cytotoxicity than did SN-38, which was used as a payload in ADC. As expected, A7R-ADC-MMAE showed a strong anti-inflammatory effect in the mouse arthritis model. These results indicate that the cytotoxicity and immunosuppression of A7R-ADC could be modulated to address the individual types or activities of the malignancies or autoimmune diseases with an appropriate payload. Exatecan, being 10-fold more potent than SN-38^59^, and the potent anti-inflammatory drug JAK inhibitor^60^ might be suitable payloads of A7R-ADC in the future.

In conclusion, we confirmed the relationship between enhanced IL-7R signaling, tumor aggressiveness and steroid-resistance in lymphoid malignancies. Moreover, enhanced IL-7R signaling also contributed to cell survival in response to steroid treatment under both physiological immune conditions and pathological autoimmune conditions. Cytotoxic agents delivered by A7R can efficiently eliminate these steroid-resistant cells, even if they have ligand-independent or autoactivated IL-7R signaling. Unlike neutralizing mAbs or other ADCs, strong or specific elimination of the malignant or autoimmune-reactive cells by A7R-ADC could restore the host immune homeostasis that was disrupted by increased abnormal IL-7R-positive cells^21^. Thus, A7R-ADC may be a promising strategy to treat malignancies and autoimmune diseases and may serve as a novel alternative to steroid therapy. We have begun evaluating the effectiveness of A7R-ADC in the treatment of other autoimmune or inflammatory diseases involving IL-7R signaling in a preclinical setting for general use. IL-7R-positive metastatic solid tumors may also be promising therapeutic targets of A7R-ADC, which should be clarified by further investigations. Finally, we are planning to advance A7R-ADC to clinical study.

## Methods

### Mouse models

The study was approved by the Committees for Animal Experimentation of the National Cancer Center. All experimental protocols and animal procedures were performed in compliance with the Guidelines for the Care and Use of Experimental Animals established by the Committee for Experimental Animals of the National Cancer Center. These guidelines meet the ethical standards required by law and comply with the guidelines for the use of experimental animals in Japan.

Female BALB/c nude mice (5 weeks old) were purchased from SLC Japan (Shizuoka, Japan) or Charles River Laboratories Japan Inc. (Yokohama, Japan). The mice were subcutaneously inoculated in their flanks with 5 × 10^6^ SupT1, Nalm6 or H2009 cells in 50 μl of Opti-MEM I (Life Technologies) and 50 μl of Growth Factor Reduced Matrigel Matrix (BD Biosciences) or 2 × 10^6^ parental or steroid-resistant CYG82 cells in 100 μl of PBS. The length (L) and width (W) of each tumor mass were measured every four or two days, and the tumor volume was calculated according to the formula (L × W^2^)/2. When the mean CYG82 tumor volume reached approximately 50 mm^3^, the mice were randomly divided into groups of 5 mice. Before the randomization, some mice with too large or small tumors were excluded. Anti-IL-7R or isotype control ADC (50 mg/kg; equivalent to an SN-38 dose of 0.6 mg/kg), anti-IL-7R antibody (50 mg/kg as an equivalent antibody dose), CPT-11 (20 mg/kg; equivalent to an SN-38 dose of 11.6 mg/kg), DEX (10 mg/kg) or saline was administered on days 0, 4, and 8 through tail vein injection. CAPE (20 mg/kg) was administered on days 0, 4, and 8 through intraperitoneal injection.

For the evaluation of axillary LN infiltration, 5 × 10^5^ CYG82 cells were inoculated into the right front food pad. The LNs were excised on day 16 after the inoculation, and the size was measured as described as above.

A CAIA mouse model was used as previously reported^34^. Briefly, female DBA/1J mice (6 weeks old, Charles River Laboratories Japan Inc.) were used. Anti-collagen 2 antibodies (Chondrex, Redmond, WA, USA) were intraperitoneally administered on day 0 at 2 mg, and 50 μg of LPS (Chondrex) was intraperitoneally injected on day 3. Before start of the treatment, the mice were randomly divided into groups of 5 mice. No mice were excluded from the experiments. Anti-IL-7R or control ADC (30 mg/kg; equivalent to an MMAE dose of 0.6 mg/kg), free MMAE (0.6 mg/kg), anti-IL-7R antibody (30 mg/kg), DEX (10 mg/kg), ETA (30 mg/kg) or saline was intraperitoneally administered on day 5. The arthritis score was calculated according to the manufacturer's instructions (maximum score per mouse = 16).

The investigators were not blinded for experiments. Sample sizes with sufficient statistic powers were carefully chosen based on estimates from pilot examinations and for appropriate assumption of normality. No statistical methods were used to predetermine sample size, but variance between comparison groups was verified to be similar.

### Cells, antibodies and reagents

The human T cell lymphoblast lymphoma cell line SUP-T1, human metastatic lung cancer cell lines H2009 and H358, human metastatic pharyngeal carcinoma cell line Detroit 562, human metastatic prostate cancer cell line PC-3, and human urinary bladder cancer cell line TCCSUP were purchased from the American Type Culture Collection (Rockville, MD, USA). The human acute lymphoblastic leukemia cell line NALM6, the human metastatic pancreatic cancer cell line PK45H and the lung cancer cell line LC-2/ad were obtained from the Cell Bank RIKEN BioResource Center (Ibaraki, Japan). Original cell lines were newly-obtained from the cell banks and used for this study. The mouse B cell leukemia cell line CYG82 and IL-7-dependent mouse pro-B cell line RAG2−/− were established as previously reported^30–32, 61^. Cells with IL-7R gene expression KD and control cells were established according to a previously reported protocol^62^. Briefly, a shRNA lentiviral expression vector targeted to human IL-7R for SUP-T1, NALM6, H2009 or PK45H cells; mouse IL-7R for CYG82 cells; or GFP as a control for SUPT1, NALM6 or CYG82 cells; or a non-specific shRNA lentiviral expression vector for H2009 or PK45H cells was used according to the manufacturer's instructions (Sigma-Aldrich Co., St. Louis, MO, USA). Mycoplasma contamination was checked by using Venor GeM Classic (Minerva Biolabs GmbH, Berlin, Germany).

The anti-mouse IL-7R monoclonal antibody (clone A7R34) and isotype control (rat IgG2a) was prepared as previously reported^12, 30, 63^. A7R34 is also commercially available (eBioscience, San Diego, CA, USA; BioLegend San Diego, CA, USA; TONBO Biosciences, San Diego, CA, USA; BD Biosciences, Franklin Lakes, NJ, USA; Abcam, Cambridge, UK; Bio-XCell, West Lebanon, NH, USA) and has been widely used in both *in vitro* and *in vivo* study^53, 64–66^. The anti-mouse CD19 monoclonal antibody (clone 1D3) was purchased from Bio-XCell. Both A7R34 and 1D3 antibody showed the same standard usage of 0.5–1μg pre 10^6^ cells as an indicator of binding activity in the flow cytometric analysis. SN-38 (Tokyo Chemical Industry Co., Tokyo, Japan) and MMAE (Medchem Express, NJ, USA) were used for the ADC. CPT-11 (irinotecan, Yakult, Tokyo, Japan), DEX (Aspen Japan KK, Tokyo, Japan), TNF-antagonist etanercept (ETA) (Pfizer, New York, NY USA) or CAPE (Selleckchem.com, Houston, TX, USA) were used in the *in vitro* and *in vivo* experiments.

### Antibody-drug conjugates (ADCs)

ADCs were produced as previously reported^33, 34, 63^. Briefly, the linker and the drug were composed of a maleimide as a connector to the mAb, Mal‐PEG_12_‐OSu (Quanta Biodesign, San Diego, CA, USA or IRIS Biotech GMBH, Marktredwitz, German) to increase polarity, and a carbamate bond or a valine‐citrulline (Val‐Cit) dipeptide to trigger cleavage by intracellular proteases. A para-amino benzyl carbamate (PABC) was used as a self-immolative spacer to efficiently release MMAE. SN-38 and MMAE were dissolved in dimethyl sulfoxide (10 mM) and stored at −80 °C. The ADCs were produced after reducing the inter-chain disulfide bonds. The DAR of the ADCs was 4–6, as determined by the colorimetric measurement of thiol groups in biological samples using Ellman’s reagent (DTNB).

The kinetics of the release of SN-38 from A7R-SN-38 or CPT-11 was investigated as previously reported^33, 34^. The samples were analyzed by high-performance liquid chromatography (HPLC) using an Alliance Waters 2795 system (Waters, Milford, MA, USA).

### Flow cytometric analysis

Flow cytometric analysis was performed as previously described^62, 67^. Alexa Fluor-647 was conjugated to each antibody according to the manufacturer’s instructions (Thermo Fisher Scientific, Waltham, MA, USA) as previously described^34^. Fluorescent antibody to CD3 (17A2, BioLegend San Diego, CA, USA; or TONBO Biosciences, San Diego, CA, USA), CD4 (GK1.5, eBioscience, San Diego, CA, USA; or BioLegend), CD8 (53–6.7, BioLegend or TONBO), CD127 (SB/199 or eBioRDR5, eBioscience; or R34-R34, TONBO), B220 (RA3–6B2, BioLegend or TONBO), IgM (RMM-1, BioLegend), common-γ (TUGm2, BioLegend) and CRLF2 (FAB5461P, R&D Systems, Minneapolis, MN, USA) were used for cell staining. To detect phospho-STAT5 and phospho-AKT, anti-phospho-STAT5 antibody (SRBCZX, eBioscience) and anti-phospho-AKT antibody (SDRNR, eBioscience) was used respectively. The stained cells were analyzed using a Guava easyCyte 10HT (Merck Millipore Co., Darmstadt, Germany) or Aria flow cytometer (BD Biosciences, Franklin Lakes, NJ, USA). Dead cells, which were stained using propidium iodide (PI) (Thermo Fisher), were excluded from the analysis. The data were analyzed using the FlowJo program (Tree Star, Ashland, OR, USA).

### Real-time quantitative RT-PCR

RNA extraction and RT-qPCR were conducted as previously described^62^. Briefly, the PCR reaction consisted of 10 µl TaqMan Fast Universal PCR Master Mix (Thermo Fisher Scientific), 1 µl TaqMan primers/probe mixture (Thermo Fisher Scientific) and 9 µl diluted template cDNA in a total volume of 20 µl. Real-time PCR was performed on an Applied Biosystems 7500 Fast System (Thermo Fisher Scientific). The relative quantification of the total RNA in each sample was conducted using the comparative Ct (threshold cycle) method. The relative expression of each gene was normalized against human GAPDH or mouse β-actin expression. All experiments consisted of at least three biological replicates and were independently performed at least two times.

### *In vivo* imaging and immunohistochemistry

*In vivo* imaging and immunostaining were performed as previously described^33, 34^. Briefly, *in vivo* or *ex vivo* fluorescence imaging was performed using an OV110 small animal imaging system (Olympus Corp., Tokyo, Japan). An Alexa-647-labeled anti-mouse IL-7R antibody (clone A7R34) or isotype control (rat IgG2a) was added to cultured cells or administered to mice. Lysotracker Red DND-99 (Thermo Fisher Scientific) was used to visualize lysosomes *in vitro*. For immunohistochemistry, the samples were incubated with an anti-IL-7R polyclonal antibody (LS-B2830/LS-C331370, LS Bio, Seattle, WA), phospho-p65/NF-κB antibody (ab86299, Abcam) or Alexa-488/555/647-labeled anti-mouse/rat/rabbit IgG (Thermo Fisher Scientific). The nuclei were visualized using DAPI (Thermo Fisher Scientific). CPT-11 has autofluorescence within the UV spectrum. Images were obtained using an LSM 710 laser scanning microscopic system (Carl Zeiss, Oberkochen, Germany) or a BZ-9000 digital high-definition microscopic system (Keyence Co., Osaka, Japan).

### *In vitro* cell cytotoxicity assay

The cytotoxicity of the cells treated with each drug for 72 hours was evaluated using a WST-8 assay (Cell Counting Kit-8, Dojindo, Kumamoto, Japan). The reaction signals were evaluated by measuring the absorbance at 450 nm using a microplate reader (SpectraMax 190, Molecular Devices Corp., Sunnyvale, CA, USA). All experiments consisted of at least three biological replicates and were independently performed at least three times.

### Gene expression analysis

Gene expression in cell lines was examined using the online databases Oncomine and Gene Expression Omnibus (GEO) and real-time quantitative RT-PCR as described above.

For the clinical analysis of raw data, CEL files of relevant datasets (GEO; GSE39339, GSE32962, GSE1919, GSE55457, Array Express; E-MEXP-3916) were downloaded. The data were normalized using the RMA procedure as previously reported^62^. Probe identification and annotation were conducted by using NetAffx, EMBL-EBI, GEO and PubMed databases^67^.

### Tissue specific expression of protein

Protein expression levels of IL-7R, CD19, CD22, CD3e, HER2 and EGFR across tissues were obtained from the PaxDB^68^.

### Statistical analysis

Significant differences between groups were determined using Student’s t-test (Figs 1b and f; 2a–c; 4c and e; 5b,c,e,f,i–l) or ANOVA (Figs 1c,d, 4a,b, 5a,m, and 6c). All analyses were performed using SPSS software version 20 (IBM, Armonk, NY, USA).

### Data availability

The data that supports the findings of this study are available from the corresponding author upon reasonable request. Gene expression data can be found at GEO (https://www.ncbi.nlm.nih.gov/geo/; GSE39339, GSE32962, GSE1919 or GSE55457), Array Express (https://www.ebi.ac.uk/arrayexpress; E-MEXP-3916) or PaxDB (http://pax-db.org/; IL-7R, CD19, CD22, CD3e, HER2, EGFR of H. sapiens).

## Electronic supplementary material

Supplementary information

## References

[1] Wang X (2016). Cancer Moonshot 2020: a new march of clinical and translational medicine. Clinical and translational medicine.

[2] Postow MA, Callahan MK, Wolchok JD (2015). Immune Checkpoint Blockade in Cancer Therapy. Journal of clinical oncology: official journal of the American Society of Clinical Oncology.

[3] Chrousos, Pavlaki, A. N. & Magiakou, M. A. In Endotext. (eds L. J. De Groot *et al*.) (MDText.com, Inc., South Dartmouth MA; 2000).

[4] Quax RA (2013). Glucocorticoid sensitivity in health and disease. Nature reviews. Endocrinology.

[5] Shah DS, Kumar R (2013). Steroid resistance in leukemia. World journal of experimental medicine.

[6] Li Y (2016). IL-7 Receptor Mutations and Steroid Resistance in Pediatric T cell Acute Lymphoblastic Leukemia: A Genome Sequencing Study. PLoS medicine.

[7] Ceredig R, Rolink AG (2012). The key role of IL-7 in lymphopoiesis. Seminars in immunology.

[8] Mazzucchelli RI, Riva A, Durum SK (2012). The human IL-7 receptor gene: deletions, polymorphisms and mutations. Seminars in immunology.

[9] Tal N, Shochat C, Geron I, Bercovich D, Izraeli S (2014). Interleukin 7 and thymic stromal lymphopoietin: from immunity to leukemia. Cellular and molecular life sciences: CMLS.

[10] Iolyeva M (2013). Interleukin-7 is produced by afferent lymphatic vessels and supports lymphatic drainage. Blood.

[11] Miller CN (2013). IL-7 production in murine lymphatic endothelial cells and induction in the setting of peripheral lymphopenia. International immunology.

[12] Sudo T (1993). Expression and function of the interleukin 7 receptor in murine lymphocytes. Proceedings of the National Academy of Sciences of the United States of America.

[13] Maki K (1996). Interleukin 7 receptor-deficient mice lack gammadelta T cells. Proceedings of the National Academy of Sciences of the United States of America.

[14] Akashi K, Kondo M, von Freeden-Jeffry U, Murray R, Weissman IL (1997). Bcl-2 rescues T lymphopoiesis in interleukin-7 receptor-deficient mice. Cell.

[15] Zenatti PP (2011). Oncogenic IL7R gain-of-function mutations in childhood T-cell acute lymphoblastic leukemia. Nature genetics.

[16] Ming J, Zhang Q, Qiu X, Wang E (2009). Interleukin 7/interleukin 7 receptor induce c-Fos/c-Jun-dependent vascular endothelial growth factor-D up-regulation: a mechanism of lymphangiogenesis in lung cancer. European journal of cancer (Oxford, England: 1990).

[17] Al-Rawi MA, Rmali K, Watkins G, Mansel RE, Jiang WG (2004). Aberrant expression of interleukin-7 (IL-7) and its signalling complex in human breast cancer. European journal of cancer (Oxford, England: 1990).

[18] Suzuki K (2013). Clinical impact of immune microenvironment in stage I lung adenocarcinoma: tumor interleukin-12 receptor beta2 (IL-12Rbeta2), IL-7R, and stromal FoxP3/CD3 ratio are independent predictors of recurrence. Journal of clinical oncology: official journal of the American Society of Clinical Oncology.

[19] Park JH (2010). Signaling by intrathymic cytokines, not T cell antigen receptors, specifies CD8 lineage choice and promotes the differentiation of cytotoxic-lineage T cells. Nature immunology.

[20] Kimura MY (2013). IL-7 signaling must be intermittent, not continuous, during CD8(+) T cell homeostasis to promote cell survival instead of cell death. Nature immunology.

[21] Park JH (2004). Suppression of IL7Ralpha transcription by IL-7 and other prosurvival cytokines: a novel mechanism for maximizing IL-7-dependent T cell survival. Immunity.

[22] Watanabe M (1998). Interleukin 7 transgenic mice develop chronic colitis with decreased interleukin 7 protein accumulation in the colonic mucosa. The Journal of experimental medicine.

[23] Penaranda C (2012). IL-7 receptor blockade reverses autoimmune diabetes by promoting inhibition of effector/memory T cells. Proceedings of the National Academy of Sciences of the United States of America.

[24] Lee LF (2012). Anti-IL-7 receptor-alpha reverses established type 1 diabetes in nonobese diabetic mice by modulating effector T-cell function. Proceedings of the National Academy of Sciences of the United States of America.

[25] McKinney, E.F. *et al*. A CD8+ T cell transcription signature predicts prognosis in autoimmune disease. *Nature medicine* **16**, 586–591, 581p following 591 (2010). 10.1038/nm.2130 PMC3504359 20400961

[26] Hartgring SA (2010). Blockade of the interleukin-7 receptor inhibits collagen-induced arthritis and is associated with reduction of T cell activity and proinflammatory mediators. Arthritis and rheumatism.

[27] Peters, C. & Brown, S. Antibody-drug conjugates as novel anti-cancer chemotherapeutics. *Bioscience reports* **35** (2015). 10.1042/BSR20150089 PMC4613712 26182432

[28] Verma S (2012). Trastuzumab emtansine for HER2-positive advanced breast cancer. The New England journal of medicine.

[29] Samuels AL (2014). A pre-clinical model of resistance to induction therapy in pediatric acute lymphoblastic leukemia. Blood cancer journal.

[30] Yasunaga M, Wang F, Kunisada T, Nishikawa S, Nishikawa S (1995). Cell cycle control of c-kit+IL-7R+ B precursor cells by two distinct signals derived from IL-7 receptor and c-kit in a fully defined medium. The Journal of experimental medicine.

[31] Yasunaga M, Adachi S, Itoh N, Nishikawa S (1995). Making the *in-vitro* model closer to actual B lymphopoiesis in the bone marrow. Seminars in immunology.

[32] Yasunaga M (1996). Involvement of Fyn tyrosine kinase in progression of cytokinesis of B lymphocyte progenitor. The Journal of cell biology.

[33] Yasunaga M, Manabe S, Tarin D, Matsumura Y (2011). Cancer-stroma targeting therapy by cytotoxic immunoconjugate bound to the collagen 4 network in the tumor tissue. Bioconjugate chemistry.

[34] Yasunaga M, Manabe S, Tarin D, Matsumura Y (2013). Tailored immunoconjugate therapy depending on a quantity of tumor stroma. Cancer science.

[35] Park MH, Kang DW, Jung Y, Choi KY, Min do S (2013). Caffeic acid phenethyl ester downregulates phospholipase D1 via direct binding and inhibition of NFkappaB transactivation. Biochemical and biophysical research communications.

[36] Puel A, Leonard WJ (2000). Mutations in the gene for the IL-7 receptor result in T(−)B(+)NK(+) severe combined immunodeficiency disease. Current opinion in immunology.

[37] Hartgring SA (2009). Elevated expression of interleukin-7 receptor in inflamed joints mediates interleukin-7-induced immune activation in rheumatoid arthritis. Arthritis and rheumatism.

[38] Hillen MR (2015). The Additive Inflammatory *In Vivo* and *In Vitro* Effects of IL-7 and TSLP in Arthritis Underscore the Therapeutic Rationale for Dual Blockade. PloS one.

[39] Senter PD, Sievers EL (2012). The discovery and development of brentuximab vedotin for use in relapsed Hodgkin lymphoma and systemic anaplastic large cell lymphoma. Nature biotechnology.

[40] Wu AM, Senter PD (2005). Arming antibodies: prospects and challenges for immunoconjugates. Nature biotechnology.

[41] Nakase K (2007). Clinical and prognostic significance of cytokine receptor expression in adult acute lymphoblastic leukemia: interleukin-2 receptor alpha-chain predicts a poor prognosis. Leukemia.

[42] Sasson SC (2010). IL-7 receptor is expressed on adult pre-B-cell acute lymphoblastic leukemia and other B-cell derived neoplasms and correlates with expression of proliferation and survival markers. Cytokine.

[43] Chen DW, Saha V, Liu JZ, Schwartz JM, Krstic-Demonacos M (2013). Erg and AP-1 as determinants of glucocorticoid response in acute lymphoblastic leukemia. Oncogene.

[44] Spijkers-Hagelstein JA (2012). Elevated S100A8/S100A9 expression causes glucocorticoid resistance in MLL-rearranged infant acute lymphoblastic leukemia. Leukemia.

[45] Jabbour E, O'Brien S, Ravandi F, Kantarjian H (2015). Monoclonal antibodies in acute lymphoblastic leukemia. Blood.

[46] Fan G, Wang Z, Hao M, Li J (2015). Bispecific antibodies and their applications. Journal of hematology & oncology.

[47] Reichert JM (2017). Antibodies to watch in 2017. mAbs.

[48] Peschon JJ (1994). Early lymphocyte expansion is severely impaired in interleukin 7 receptor-deficient mice. The Journal of experimental medicine.

[49] Maraskovsky E (1996). Impaired survival and proliferation in IL-7 receptor-deficient peripheral T cells. Journal of immunology (Baltimore, Md.: 1950).

[50] Vely F (2016). Evidence of innate lymphoid cell redundancy in humans. Nature immunology.

[51] Vivier E, van de Pavert SA, Cooper MD, Belz GT (2016). The evolution of innate lymphoid cells. Nature immunology.

[52] Klose CS, Artis D (2016). Innate lymphoid cells as regulators of immunity, inflammation and tissue homeostasis. Nature immunology.

[53] Powell N (2012). The transcription factor T-bet regulates intestinal inflammation mediated by interleukin-7 receptor+ innate lymphoid cells. Immunity.

[54] Kong FK, Chen CL, Cooper MD (2002). Reversible disruption of thymic function by steroid treatment. Journal of immunology (Baltimore, Md.: 1950).

[55] Gruver-Yates AL, Quinn MA, Cidlowski JA (2014). Analysis of glucocorticoid receptors and their apoptotic response to dexamethasone in male murine B cells during development. Endocrinology.

[56] Lee HC, Shibata H, Ogawa S, Maki K, Ikuta K (2005). Transcriptional regulation of the mouse IL-7 receptor alpha promoter by glucocorticoid receptor. Journal of immunology (Baltimore, Md.: 1950).

[57] Franchimont D (2002). Positive effects of glucocorticoids on T cell function by up-regulation of IL-7 receptor alpha. Journal of immunology (Baltimore, Md.: 1950).

[58] Martin DN, Mikhail IS, Landgren O (2009). Autoimmunity and hematologic malignancies: associations and mechanisms. Leukemia & lymphoma.

[59] Ogitani Y (2016). DS-8201a, A Novel HER2-Targeting ADC with a Novel DNA Topoisomerase I Inhibitor, Demonstrates a Promising Antitumor Efficacy with Differentiation from T-DM1. Clinical cancer research: an official journal of the American Association for Cancer Research.

[60] Schwartz DM, Bonelli M, Gadina M, O’Shea JJ (2016). Type I/II cytokines, JAKs, and new strategies for treating autoimmune diseases. Nature reviews. Rheumatology.

[61] Ogawa M, Ikuta K, Katsura Y, Nishikawa S (1989). Stepwise progression of B cell malignancy occurred in a bone marrow stromal cell-dependent pre-B cell clone. Leukemia.

[62] Yasunaga M, Matsumura Y (2014). Role of SLC6A6 in promoting the survival and multidrug resistance of colorectal cancer. Scientific reports.

[63] Koga Y (2015). Antitumor effect of antitissue factor antibody-MMAE conjugate in human pancreatic tumor xenografts. International journal of cancer. Journal international du cancer.

[64] Jin J, Goldschneider I, Lai L (2011). *In vivo* administration of the recombinant IL-7/hepatocyte growth factor beta hybrid cytokine efficiently restores thymopoiesis and naive T cell generation in lethally irradiated mice after syngeneic bone marrow transplantation. Journal of immunology (Baltimore, Md.: 1950).

[65] Mazzon C (2011). The critical role of agrin in the hematopoietic stem cell niche. Blood.

[66] Deiser K, Stoycheva D, Bank U, Blankenstein T, Schuler T (2016). Interleukin-7 Modulates Anti-Tumor CD8+ T Cell Responses via Its Action on Host Cells. PloS one.

[67] Yasunaga, M. *et al*. In Nat Biotechnol, Vol. 23 1542–1550 (2005). 10.1038/nbt1167 16311587

[68] Wang M (2012). PaxDb, a database of protein abundance averages across all three domains of life. Molecular & cellular proteomics: MCP.

