# Supplementary material for: Immunoregulation by IL-7R-targeting antibody-drug conjugates: overcoming steroid-resistance in cancer and autoimmune disease
Source: Sci Rep. 2017 Sep 6;7:10735. doi: 10.1038/s41598-017-11255-4 (PMC5587554; doi:10.1038/s41598-017-11255-4)
Supplement: Supplementary file 1 — Supplementary information [file 41598_2017_11255_MOESM1_ESM.pdf]

Supplementary information

Immunoregulation by IL-7R-targeting antibody-drug conjugates: overcoming steroid-resistance in cancer and autoimmune disease

Masahiro Yasunaga, Shino Manabe, Yasuhiro Matsumura

Supplementary Figure Legends

Supplementary Figure 1. Microarray analyses of gene expression in acute lymphoblastic leukemia.

Gene expression (arbitrary units) of TdT, CD19, CD22 or IL-7R in B cell acute lymphoblastic leukemia (B-ALL), obtained from the GSE39339 (top) or GSE32962 (middle) dataset, and of CD2, CD3E, CD5, CD7 or IL-7R in T cell acute lymphoblastic leukemia (T-ALL), obtained from the E-MEXP-3916 dataset (bottom). Pre, pre-treatment; Post, post-treatment; S, steroid sensitive; R, steroid resistant; N, no treatment; T, treatment. *$P<0.05$, ***$P<0.001$. Significant differences between the groups were determined using Student's t-test (SPSS software version 20, IBM).

Supplementary Figure 2. Evaluation of immunosuppression caused by A7R-ADC or steroid treatment.

(Top) Changes in cell numbers in response to A7R-ADC-SN-38 treatment (0.6 mg/kg as an equivalent SN-38 dose) or high-dose DEX (10 or 40 mg/kg) at 7 days after injection. Thy, thymus; BM, bone marrow; Sp, spleen. Each bar represents the mean±SD (n=3). *$P<0.05$, **$P<0.01$, ***$P<0.001$. Significant differences between the groups were determined using Student's t-test (SPSS software version 20, IBM).

(Bottom) Flow cytometric analysis of IL-7R, CD3, CD4, CD8, B220, IgM, common-γ and

CRLF2 was performed in the cells shown in (A).

Supplementary Figure 3. Microarray analyses of gene expression in rheumatoid arthritis.

Gene expression (arbitrary units) of IL-7R in synovial tissues of patients with rheumatoid arthritis, obtained from the GSE1919 (left) or GSE55457 (right) dataset. Pre, pre-treatment; Post, post-treatment; S, steroid sensitive; R, steroid resistant; N, no treatment; T, treatment. *P<0.05, **P<0.01, ***P<0.001. Significant differences between the groups were determined using Student's t-test (SPSS software version 20, IBM).

Supplementary Figure 4. Anti-inflammatory effect of A7R-ADC-SN-38 in mouse autoimmune arthritis.

Kinetics of arthritis scores in mice with CAIA were evaluated with saline as a control, DEX (10 mg/kg), A7R (50 mg/kg as an equivalent antibody dose of ADCs), ADC with SN-38 (ACD19-SN-38, 50 mg/kg) or A7R-SN-38 (50 mg/kg, both ADCs; 0.6 mg/kg as an equivalent SN-38 dose) (n=5). P<0.01 (saline or A7R vs. A7R-SN-38; DEX vs. ACD19-SN-38), P<0.001 (saline or A7R vs. DEX). Bar=SD.

Significant differences between the groups were determined using ANOVA (SPSS software

version 20, IBM).

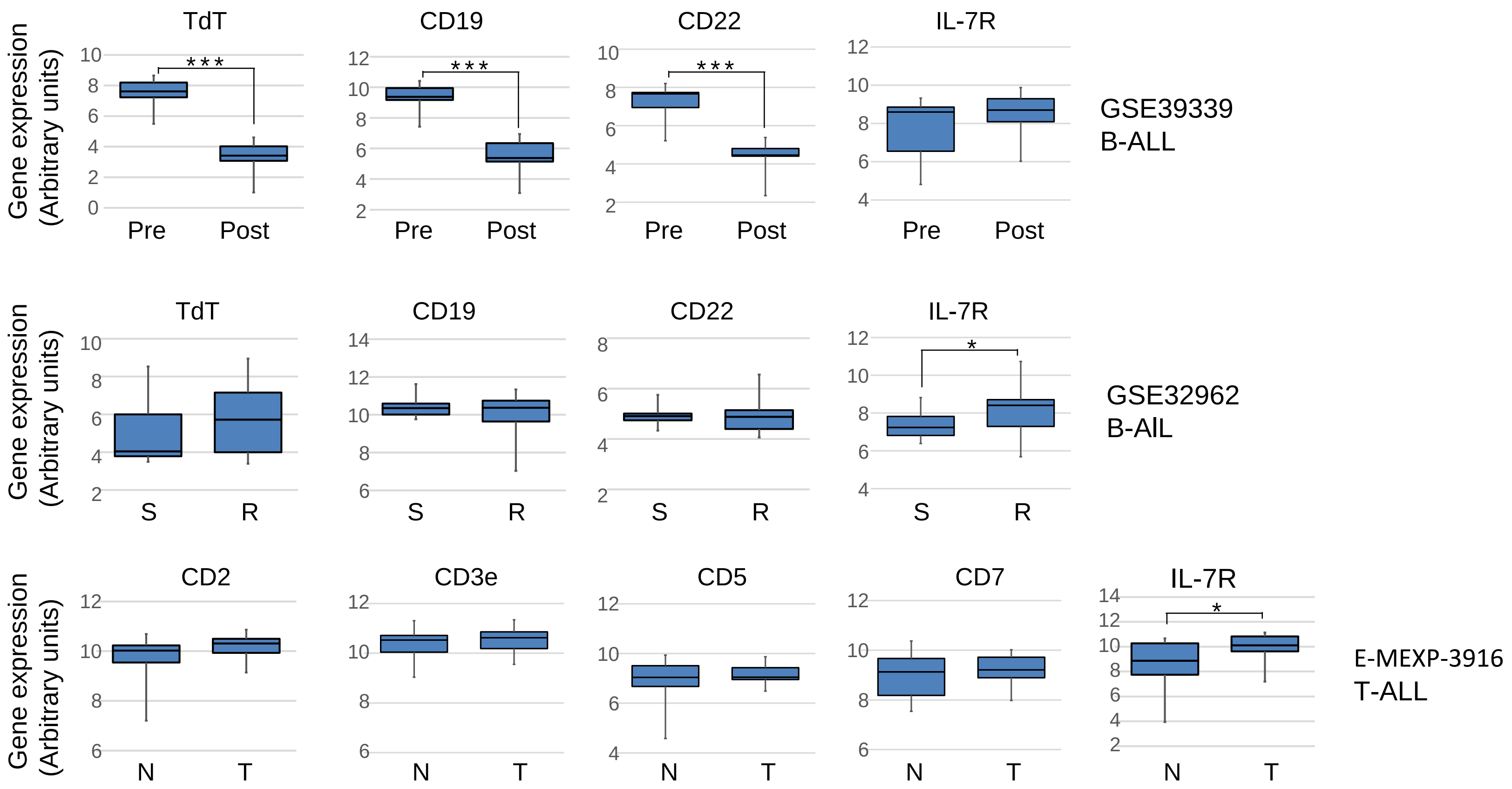

Supplementary Figure 1

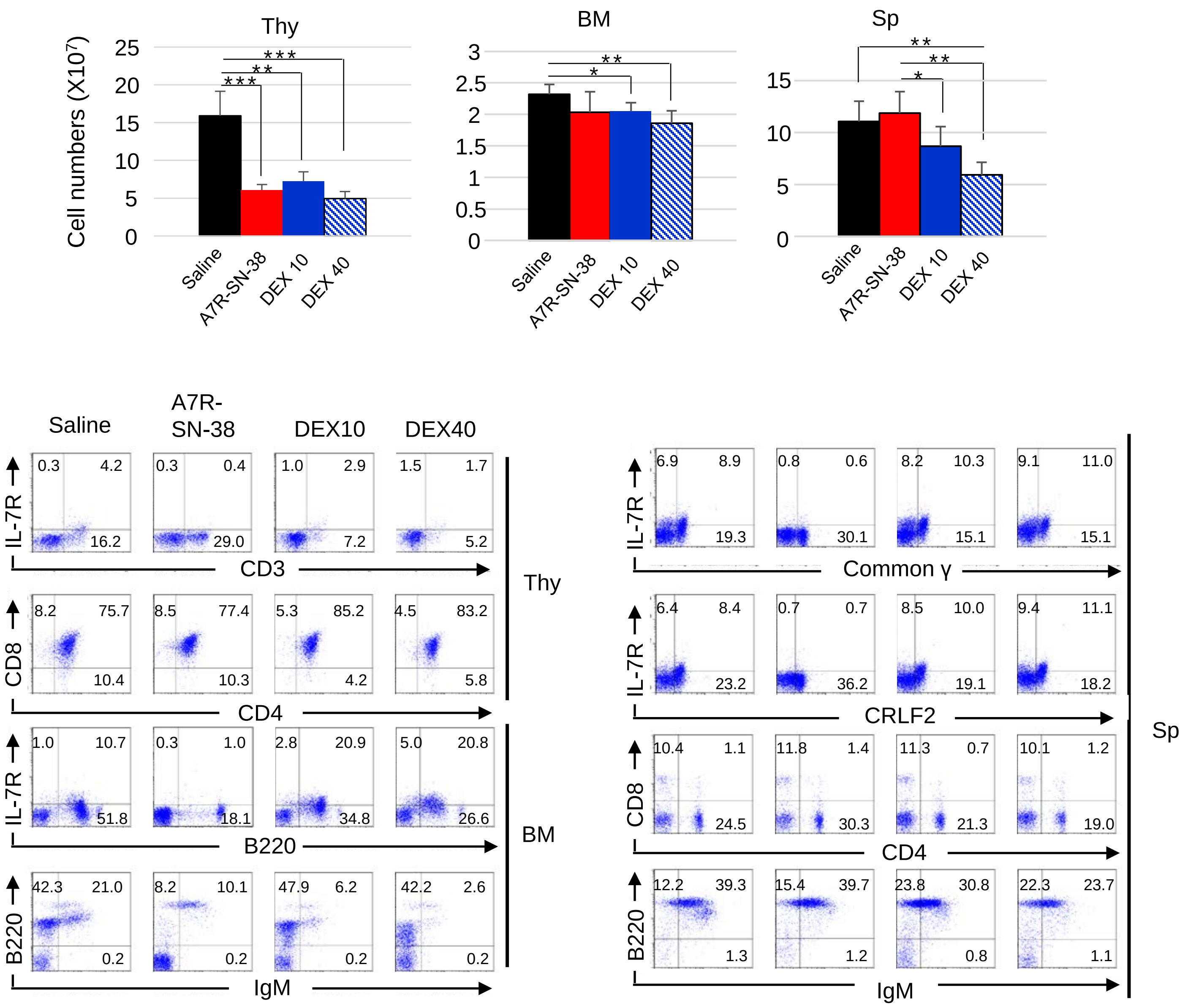

Supplementary Figure 2

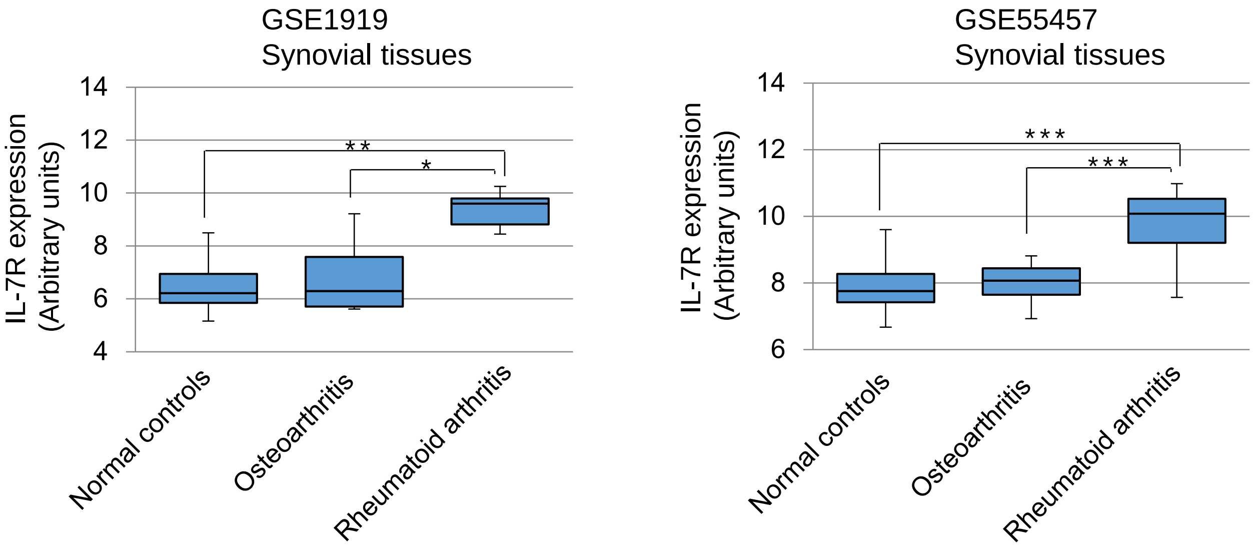

Supplementary Figure 3

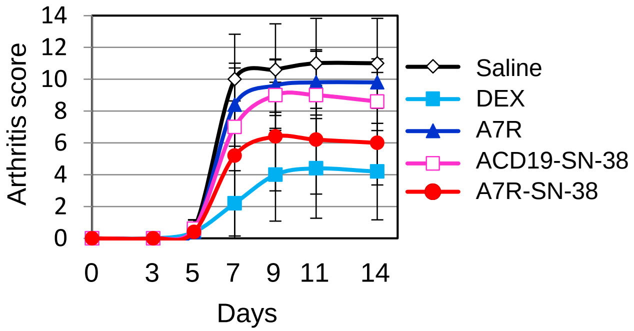

Supplementary Figure 4

Supplementary Table 1 **Tissue specific expression of protein**

Tissue name	Data Origin	Protein name					
		IL7R	CD19	CD22	CD3e	HER2	EGFR
CD4 T cells	Kim, Nature, 2014	40.8			561		
CD8 T cells	Kim, Nature, 2014	8.13			657		
CD20 B cells	Kim, Nature, 2014	0.69	132	257	103		0.04
CD56 NK cells	Kim, Nature, 2014	0.33		2.45	144		
Lymph node	Wilhelm Nature 2014		30.1	62.9	351		1.15
Spleen	Wilhelm Nature 2014					1.29	11.4
Brain	Kim, Nature, 2014			0.45		1.2	39.7
Brain	Wilhelm Nature 2014					1.38	37.5
Brain	Integrated					0.07	2.71
Brain	PeptideAtlasAug2013						0.95
Heart	PeptideAtlasAug2014					3.19	4.37
Heart	Kim, Nature, 2014						0.74
Heart	Integrated				0.3	0.48	0.72
Lung	Wilhelm Nature 2014					1.91	30.2
Lung	Kim, Nature, 2014				25.6		15.2
Lung	Integrated				3.2	0.24	5.68
Liver	PeptideAtlasAug2013						3.76
Liver	Kim, Nature, 2014				3.27	2.34	50.1
Liver	Wilhelm Nature 2014				18.6	6.18	20.5
Liver	Integrated		4.11	7.67	0.62	1.34	6.72
Liver	DLPEP2006					1.77	5.88
Liver	DLPEP2007		8.22	3.15			2.81
Liver	CHLPPC,J.Proteome_Res,2010			48.7			
Kidney	Wilhelm Nature 2014					2.21	12.5
Kidney	Integrated				1.49	1.1	7.43
Kidney	Kim, Nature, 2014				5.96		2.96
Kidney	PeptideAtlasAug2013						1.73
Pancreas	Kim, Nature, 2014				39.8	5.31	16
Pancreas	Integrated				19.9	3.35	13
Pancreas	Wilhelm Nature 2014					1.4	10
Adrenal grand	Kim, Nature, 2014				18.3	2.12	6.27
Urinary bladder	Kim, Nature, 2014				2.26	2.56	13.4
Prostate	Wilhelm Nature 2014					0.77	22.9
Prostate	Integrated		0.1		1.26	5.09	19.1
Prostate	Kim, Nature, 2014		0.1		2.52	9.41	15.3
Testis	Kim, Nature, 2014	0.38			8.55	0.94	25.1
Testis	Wilhelm Nature 2014					2.6	21.1
Testis	Integrated	0.1		0.04	2.88	2.9	19.8
Female Gonad	Kim, Nature, 2014	0.3		3.68	4.75	3.99	30.4
Female Gonad	Integrated	0.08		0.92	2.34	1.81	19.7
Fallopain tube	Wilhelm Nature 2014					1.19	29.5
Uterine cervix	Wilhelm Nature 2014					1.82	28.6
Uterine	Integrated					2.21	
Skin	L Amon, FHCRC, ID18251-18264					6.95	46.8
Skin	Integrated					3.48	23.4
Retina	Kim, Nature, 2014					3.67	40.7
Saliva	Wilhelm Nature 2014					4.68	31.8
Saliva	Integrated					2.34	15.9
Saliva secreting gland	Wilhelm Nature 2014					3.78	29
Oral cavity	Wilhelm Nature 2014				34.2	0.99	19.8
Esophagus	Wilhelm Nature 2014					3.74	72.2
Esophagus	Integrated				2.11	1.87	49.7
Esophagus	Kim, Nature, 2014				4.22		27.2
Stomach	Wilhelm Nature 2014					2.94	25.6
Colon	Wilhelm Nature 2014					4.45	32.7
Colon	Integrated				4.79	3.54	21.9
Colon	Kim, Nature, 2014				9.57	2.64	11.1
Rectum	Kim, Nature, 2014			2.27	15		9.09
Rectum	Integrated			1.14	7.48	1	6.75
Rectum	Wilhelm Nature 2014					2	4.41

Unit; ppm
